# Therapeutic effects of antibiotics loaded cellulose nanofiber and κ-carrageenan oligosaccharide composite hydrogels for periodontitis treatment

**DOI:** 10.1038/s41598-020-74845-9

**Published:** 2020-10-22

**Authors:** Athira Johnson, Fanbin Kong, Song Miao, Hong-Ting Victor Lin, Sabu Thomas, Yi-Cheng Huang, Zwe-Ling Kong

**Affiliations:** 1 Department of Food Science, National Taiwan Ocean University, Pei-Ning Road, Keelung, 20224 Taiwan, ROC; 2 Department of Food Science and Technology, University of Georgia, 100 Cedar Street, Athens, GA 30602 USA; 3 Teagasc Food Research Centre, Moorepark, Fermoy, Co., Cork, P61 C996 Ireland; 4 School of Energy Studies and School of Chemical Sciences, Mahatma Gandhi University, Priyadarshini Hills P.O, Kottayam, Kerala 686560 India

**Keywords:** Drug discovery, Immunology, Microbiology, Diseases, Health care, Nanoscience and technology

## Abstract

Periodontitis is an inflammatory disease that can lead to the periodontal pocket formation and tooth loss. This study was aimed to develop antimicrobials loaded hydrogels composed of cellulose nanofibers (CNF) and κ-carrageenan oligosaccharides (CO) nanoparticles for the treatment of periodontitis. Two antimicrobial agents such as surfactin and Herbmedotcin were selected as the therapeutic agents and the hydrogels were formulated based on the increasing concentration of surfactin. The proposed material has high thermal stability, controlled release, and water absorption capacity. This study was proceeded by investigating the in vitro antibacterial and anti-inflammatory properties of the hydrogels. This material has strong antibacterial activity against periodontal pathogens such as *Streptococcus mutans*, *Porphyromonas gingivalis*, *Fusobacterium nucleatum*, and *Pseudomonas aeruginosa*. Moreover, a significant increase in malondialdehyde (MDA) production and a decrease in biofilm formation and metabolic activity of the bacteria was observed in the presence of hydrogel. Besides, it reduced the reactive oxygen species (ROS) generation, transcription factor, and cytokines production in human gingival fibroblast cells (HGF) under inflammatory conditions. In conclusion, the hydrogels were successfully developed and proven to have antibacterial and anti-inflammatory properties for the treatment of periodontitis. Thus, it can be used as an excellent candidate for periodontitis treatment.

## Introduction

Periodontitis is an oral disease that affects about 10–15% of the world population^1^. It is an inflammatory disease characterized by the loss of tissue attachment, gingival recession, and periodontal pocket formation^2^. The initiation of inflammation in the gingival area is due to the formation of bacterial plaque and the respective host immune response^3^. The most important pathogens associated with periodontitis are *Aggregatibacter actinomycetemcomitans*, *Prevotella intermedia*, *Tannerella forsythia*, *Parvimonas micra*, and *Porphyromonas gingivalis*^4^. During the initiation of periodontitis, the symbiotic microbial community is converted into a dysbiotic microbial community^5^. Gradually, the inflammation begins with activation of the innate immune system by toll-like receptors (TLRs) and subsequent release of pro-inflammatory cytokines and recruitment of phagocytes and lymphocytes to the affected area^6^. Moreover, certain diseases are also enhanced by periodontitis namely stroke, diabetes, atherosclerosis, chronic obstructive pulmonary disease, and coronary heart disease^7^. The progression and formation of periodontitis are depending upon some factors like smoking, age, sex, socioeconomic status, education, and genetic condition, etc.^8^. So, it is necessary to manage this disease to maintain oral health and associated disease. The primary approach to control the periodontitis is by regular brushing and flossing but it is not very effective after the progression of the disease. Tooth scaling, root planing, ultrasonic scalars, use of lasers, and the use of chemotherapeutic agents (e.g. tetracycline and minocycline), etc. are commonly used for clinical periodontitis treatment. But, the mechanical treatment is limited due to time consuming nature, difficulties to use instruments, and recolonization of bacteria, and the chemical treatment was limited due to the inability to achieve high gingival crevicular fluid concentration, adverse drug reactions, presence of multiple drug-resistant microorganism^9,10^.

To improve the drawback of these conventional treatments, different types of drug delivery systems are developed. They are designed to deliver drugs into the targeted area to retain the therapeutic effects for a long term^2^. A local drug delivery system has certain advantages like direct access, improvement of patient compliance, enhanced efficacy, painless, and more convenience in usage, etc.^11^. Hydrogels are specially designed drug delivery systems that got special attention due to the physical similarity (high water content) with tissues, biocompatibility, and controlled drug release. It is a three-dimensional polymeric network structure that extensively swollen in water and is formed by physical or chemical cross-linking^12^. They can absorb water in the presence of external stimuli such as pH, ionic strength, temperature, magnetic field, and electric field^12^. Hydrogels can be fabricated by using natural or synthetic polymers or by using the combination of both. A previous study mentioned that hydrogels have a large potential in periodontal therapy due to its semisolid nature and easy adherence to dental pocket^11^.

The current study is focusing on the application of antimicrobials loaded modified-nanocellulose based drug delivery systems to treat periodontitis. Nanocellulose has certain advantages such as high specific surface area, biodegradability, good reinforcement, easy functionalization, biocompatibility, and high elastic modulus. Generally, it is categorized into cellulose nanocrystals [length ca. 100–200 nm (CNC)], cellulose nanofibers [length > 1 μm (CNF)], and bacterial cellulose (BC)^13,14^. It was noted that in the presence of salt or under low pH conditions, CNF will readily form hydrogels via the reduction in surface charge through the interaction of counterion-driven charge screening of surface carboxyl groups^14^. Previous study showed that nanocellulose reinforced composite hydrogels were prepared by the addition of different polymers such as alginate, collagen, gelatin, poly (vinyl alcohol), Poly (ethylene glycol). In addition to this, some inorganic particles were also used to prepare a nanocellulose based hydrogel systems^13^. The present study investigated the preparation of hydrogels from κ-carrageenan oligosaccharide (CO) linked CNF nanoparticles and subsequent drug loading. Our previous study reported that the addition of CO into CNF improved the degradation temperature, crystallinity, and swelling property of CNF^15^.

Carrageenan is a carbohydrate polymer extracted from red seaweeds. It is an anionic sulfated linear polysaccharide that has a wide range of pharmacological activities such as antiviral, antitumor, anticoagulant, immunomodulatory, and antihyperlipidemic activities^16^. It is composed of alternating α-(1 → 3) and β-(1 → 4) linked d-galactosyl residues^17^. κ-carrageenan consists of d-galactose having sulfate at the C4 position linked to anhydro-galactose^16^. They can undergo gelation by the conformational change from the coil to double helices structure and subsequent aggregation^18^.

Two antimicrobials named Herbmedotcin and surfactin were selected for preparing the drug delivery system. Herbmedotcin is a safe, effective, and patented antibacterial agent formulated by using natural organic material and super quantum dots^19^. It is developed by a Taiwan based company called Giant Bio Tech^20^. This product has a large application in healthcare, agriculture, and aquaculture sectors^19,20^. The antimicrobial activity of Herbmedotcin is based on the disruption of the bacterial cell wall by its positive charge^20^. It is a safe material and its lethal dose 50 (LD50) is greater than 2000 ppm^20^. Surfactin is a biosurfactant that has been largely extracted from the microorganism called *Bacillus subtilis*. It is a cyclic lipopeptide consists of hydrophilic peptide ring (seven amino acids) and a hydrophobic fatty acid chain (13–16 carbon atoms)^21^. It is well-known for certain pharmacological properties such as antiviral, antibacterial, antifungal, anti-mycoplasma, anti-inflammatory, and thrombolytic properties^22^. Previous literature reported the inhibition of biofilm by the specific antiadhesive activity of surfactin against *Streptococcus aureus* and *Escherichia coli* on polystyrene^23^. Surfactin increases the surface pressure and alters the membrane thickness by penetrating the plasma membrane of the bacteria via hydrophobic interactions^24^. The previous study reported the inhibition of the increase in the expression of inflammatory markers such as interferon-gamma, interleukin-6 (IL-6), inducible nitric oxide synthase, and nitric oxide (NO) by surfactin under lipopolysaccharide (LPS)-induced inflammatory condition^25^. It was also noted that nanotechnology-based surfactin delivery reduced the toxicity and overcome the multi-drug resistance of the bacteria^24^.

In recent days, the demand for a biopolymer-based hydrogel drug delivery system for periodontitis is increased. Recently, Xu et al. developed an injectable thermo-responsive hydrogel composed of chitosan, β-sodium glycerophosphate, and gelatin for the delivery of aspirin and erythropoietin. This material has anti-inflammation and periodontium regeneration function^26^. However, the literature mentioned that chitosan is not strongly supported tissue regeneration as demonstrated by its effect on the width of keratinized gingiva in dogs^27^. A biomimetic collagen-sodium alginate-titanium oxide 3D matrix has been developed by Elango et al.^28^. It has been reported that this material has good water binding capacity and it promoted the differentiation of human periodontal ligament fibroblasts^28^. Although, the local drug delivery systems for periodontitis are limited due to small area, difficult to administer local irritants, presystemic metabolism by enzymes, and manufacturing cost, etc.^29,30^. The present study provides an insight into the development of novel hydrogel as potential material for the treatment of periodontitis. In this work, we evaluated the characteristics, antibacterial, and anti-inflammatory properties of surfactin and Herbmedotcin loaded CO-CNF hydrogels. The natural origin of the material helps to reduce the toxicity to the cells and it facilitated the incorporation of drugs by cross-linking. Our study revealed that obtained hydrogel has high thermal stability, water-absorbing capacity, and less toxicity. We finally demonstrated that this hydrogel has good antimicrobial activity against pathogens which cause directly or indirectly periodontitis such as *S. mutans*, *P. gingivalis*, *F. nucleatum*, and *P. aeruginosa* and strong anti-inflammatory activity under LPS-induced inflammatory condition in periodontitis.

## Results

### Scanning electron microscopic (SEM) analysis

**Figure 1:**
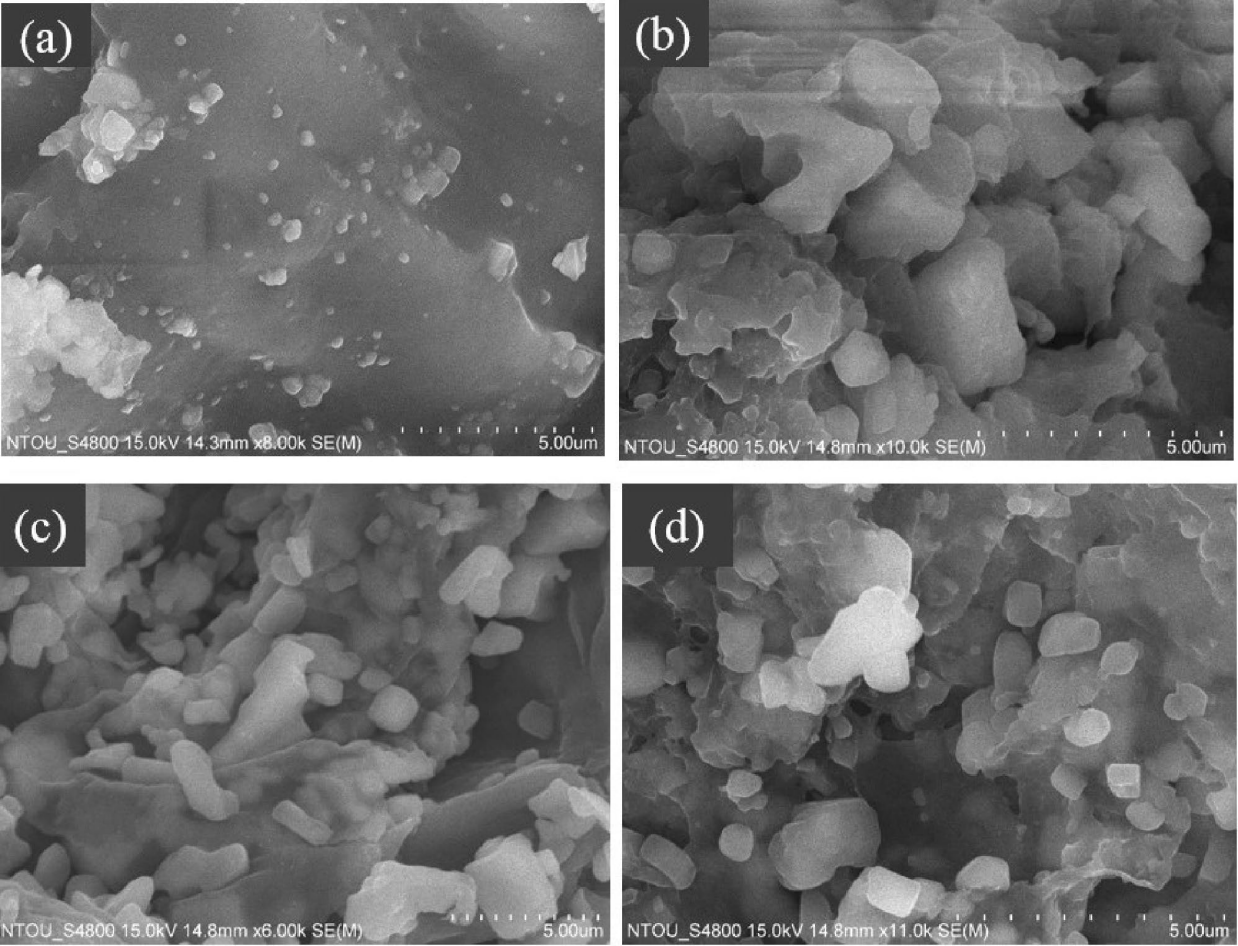
Scanning electron microscopic (SEM) images of hydrogels. Dried samples were transferred to the metal stud using double-sided tape and coated with gold. Images were observed at different magnifications at an accelerating voltage of 15 kV. The scale bar represents 5 µm. CCH: κ-carrageenan oligosaccharides linked cellulose nanofibers hydrogel, 100SH: 100 mg surfactin and Herbmedotcin loaded hydrogel, 200SH: 200 mg surfactin and Herbmedotcin loaded hydrogel, and 400SH: 400 mg surfactin and Herbmedotcin loaded hydrogel.

Antimicrobials loaded hydrogels were prepared from CO-CNF nanoparticles. The morphology of hydrogels was observed by SEM as shown in Fig. 1. In CCH, there were a lot of solid blocks that were seen on a flat plane. These blocks were more visible in 100SH, 200SH, and 400SH. These clusters might be originated from the cross-linking of CO-CNF nanoparticles in presence of *N*,*N*′-methylenebisacrylamide (MBAA). It was understood that CNF on CO-CNF has appeared as a long fiber in which CO was attached as small blocks^15^. Besides, large pore formation was also observed, especially in 400SH. This type of morphology may be due to the formation of an elastic gel by the conformational transition from random coil to double helix upon cooling and subsequent aggregation^31^.

### Fourier-transform infrared spectroscopic (FTIR) analysis

**Figure 2:**
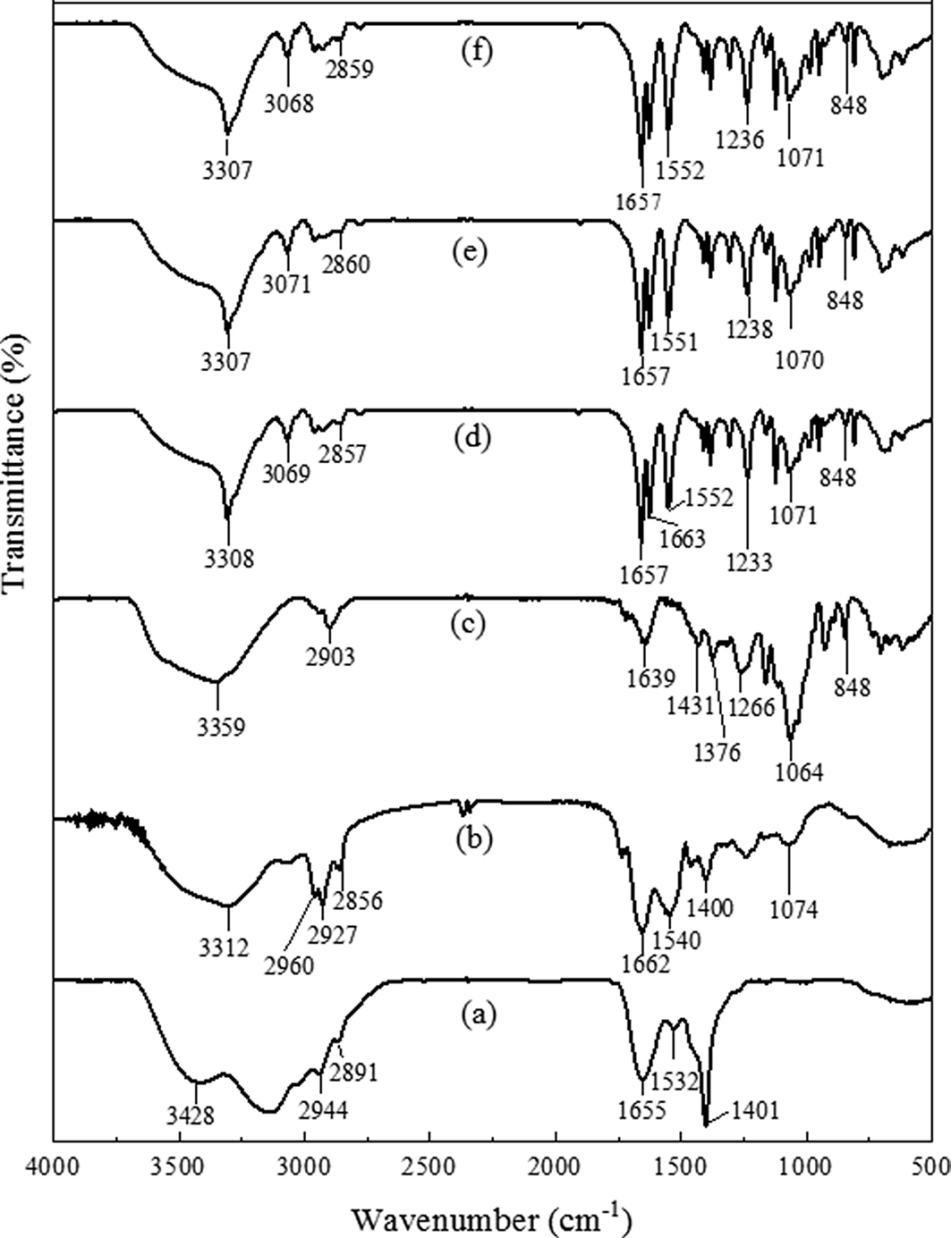
Fourier-transform infrared spectra (FTIR) of (**a**) Herbmedotcin, (**b**) Surfactin, (**c**) CCH, (**d**) 100SH, (**e**) 200SH, and (**f**) 400SH. The samples were pelletized using potassium bromide (1:100, w/w) and a total of 64 scans were performed. CCH: κ-carrageenan oligosaccharides linked cellulose nanofibers hydrogel, 100SH: 100 mg surfactin and Herbmedotcin loaded hydrogel, 200SH: 200 mg surfactin and Herbmedotcin loaded hydrogel, and 400SH: 400 mg surfactin and Herbmedotcin loaded hydrogel.

FTIR analysis of hydrogels was given in Fig. 2. Herbmedotcin is a complex mixture consisting of various bioactive components. The peak at 3428 cm^−1^ represents the presence of –OH and –NH stretching vibrations^32^. A small peak at 2944 cm^−1^ indicates the presence of alkenes and a peak at 2891 cm^−1^ corresponding to C–H stretching^33,34^. The peak at 1655 cm^−1^ represents N–H bending vibrations^32^. A sharp peak at 1401 cm^−1^ is corresponding to the –C=C– stretching vibration of carboxylic acids^35^. Another compound present in the hydrogel was surfactin. The peak at 3312 cm^−1^, 2960 cm^−1^, and 2927 cm^−1^ correspond to N–H stretching mode and lipopeptide portion of the surfactin^36^. A small peak at 2856 cm^−1^ represents the presence of an aliphatic chain and a deep peak at 1540 cm^−1^ indicates the deformation mode of the N–H bond combines with C–N stretching mode^37^. A detailed FTIR spectrum of CO-CNF was elucidated in our previously reported work^15^. CCH hydrogels were formed in the presence of an MBAA as a cross-linker. The band observed at 1639 cm^−1^ in CCH assigned to C=C stretching and presence of carboxylate ions indicated by two peaks, 1431 cm^−1^_,_ and 1376 cm^−1^ ^38^. The asymmetric stretch of O=S=O (1266 cm^−1^) and –O–SO_3_ (848 cm^−1^) were also observed. It was noted that there was no big difference between the FTIR spectra of 100SH, 200SH, and 400SH. A small sharp peak formed at 3307 cm^−1^ was an amide bond A supposed to be coming from surfactin^39^. A small speak shifting at 2856 cm^−1^ in surfactin was also noted and a peak coming from Herbmedotcin was seen at 1657 cm^−1^. A short peak at 848 cm^−1^ was supposed to be originated from CCH. So, it was understood that the antimicrobial agents were successfully entrapped in the hydrogel matrix.

### Thermogravimetric analysis (TGA)

**Figure 3:**
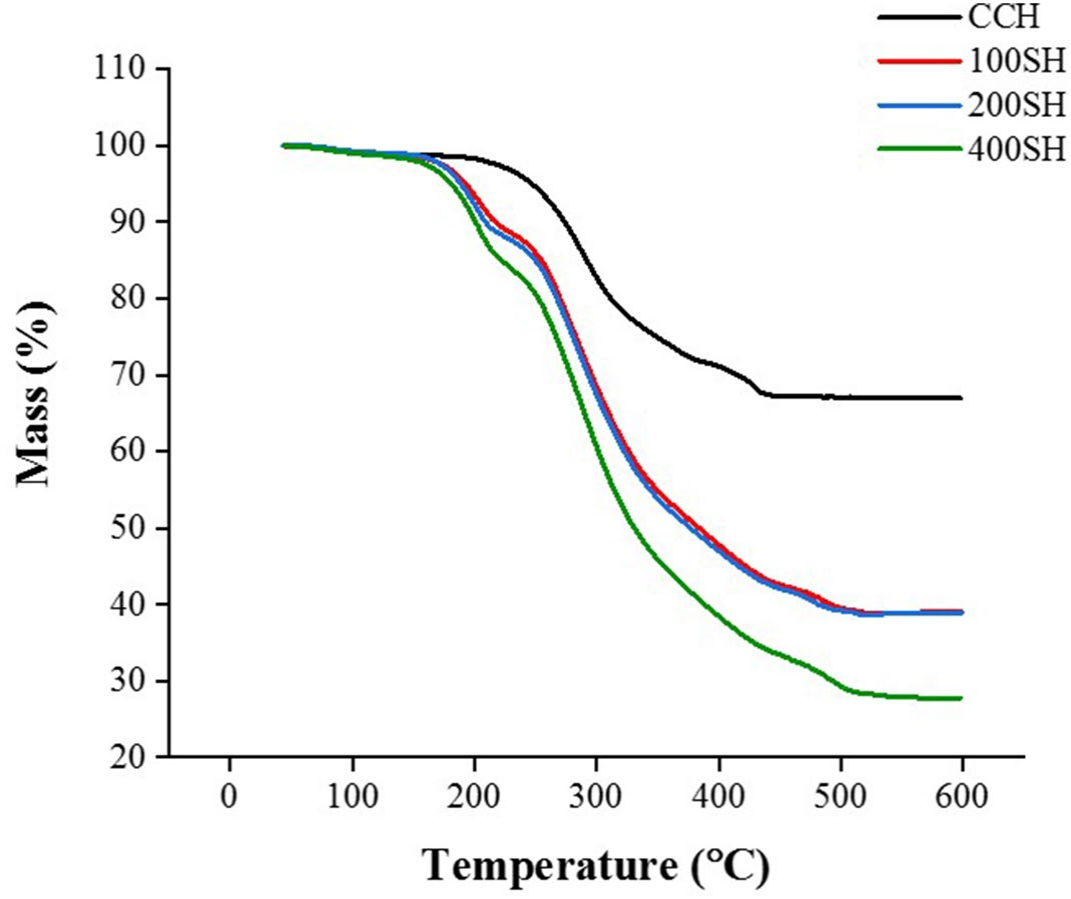
Thermogravimetric Analysis (TGA) of CCH, 100SH, 200SH, and 400SH. The temperature range was fixed from 40–600 °C with a constant heating rate of 20 °C/min under nitrogen atmosphere. CCH: κ-carrageenan oligosaccharides linked cellulose nanofibers hydrogel, 100SH: 100 mg surfactin and Herbmedotcin loaded hydrogel, 200SH: 200 mg surfactin and Herbmedotcin loaded hydrogel, and 400SH: 400 mg surfactin and Herbmedotcin loaded hydrogel.

The thermal degradation of hydrogels is given in Fig. 3. A slight reduction in weight was observed between 50 and 150 °C and this may due to the evaporation of moisture. In CCH, this weight loss was continued to 230 °C, then decreased suddenly, and finally reached to a degradation temperature at 432 °C. It was almost similar to 100H, 200SH, and 400SH with slight changes. A sharp decline in the weight loss was observed from about 166 °C in 100SH, 200SH, and 400SH and the degradation temperature of the above said hydrogels was around 510 °C.

### Entrapment efficiency (EE), loading capacity (LC) and in-vitro drug release

**Figure 4:**
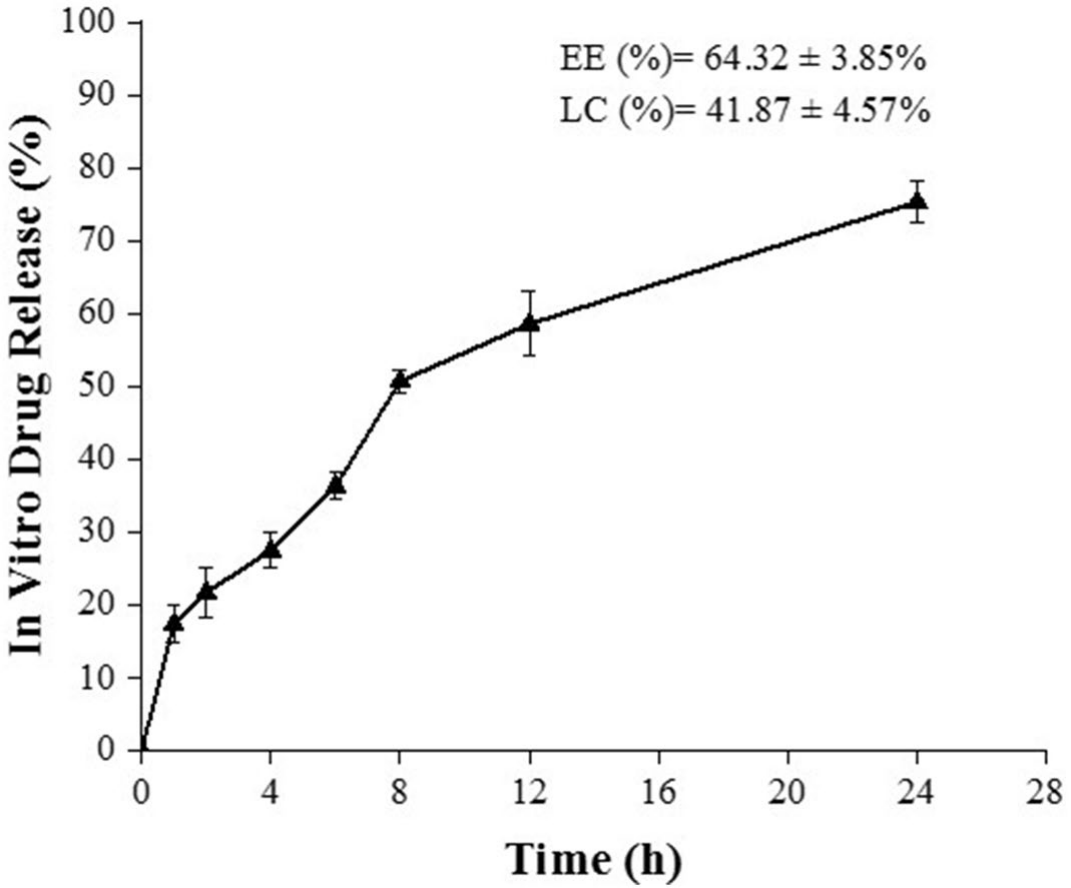
In vitro drug release profile of surfactin from the hydrogel. Release of surfactin between 0 to 24 h. EE: entrapment efficiency, LC: loading capacity.

The EE and LC of surfactin loaded-CCH was 64.32 ± 3.85% and 41.87 ± 4.57%, respectively (Fig. 4). A rapid drug release of surfactin was observed from 0 to 2 h. It might be due to the release of physically entrapped drugs. After 4 h, a controlled release of drugs was observed and about 75% of the drug was released after 24 h. The in-vitro release profile of hydrogel showed a slower controlled release of surfactin. The slower release of surfactin may due to the high cross-linking of CO-CNF with surfactin that evident from the TGA and FTIR analysis. Release of a drug from a carrier depending upon various factors. Literature mentioned that the drug release can be divided into delayed-release, site-specific targeting, receptor targeting, and sustained release. And the sustained release can be divided into controlled-release and extended-release. Among them, the extended type of drug release system has slow drug release^40^. According to our results, the material has a slow release of drug and about 50% of drugs were released after 8 h.

### Swelling ratio of hydrogels

**Figure 5:**
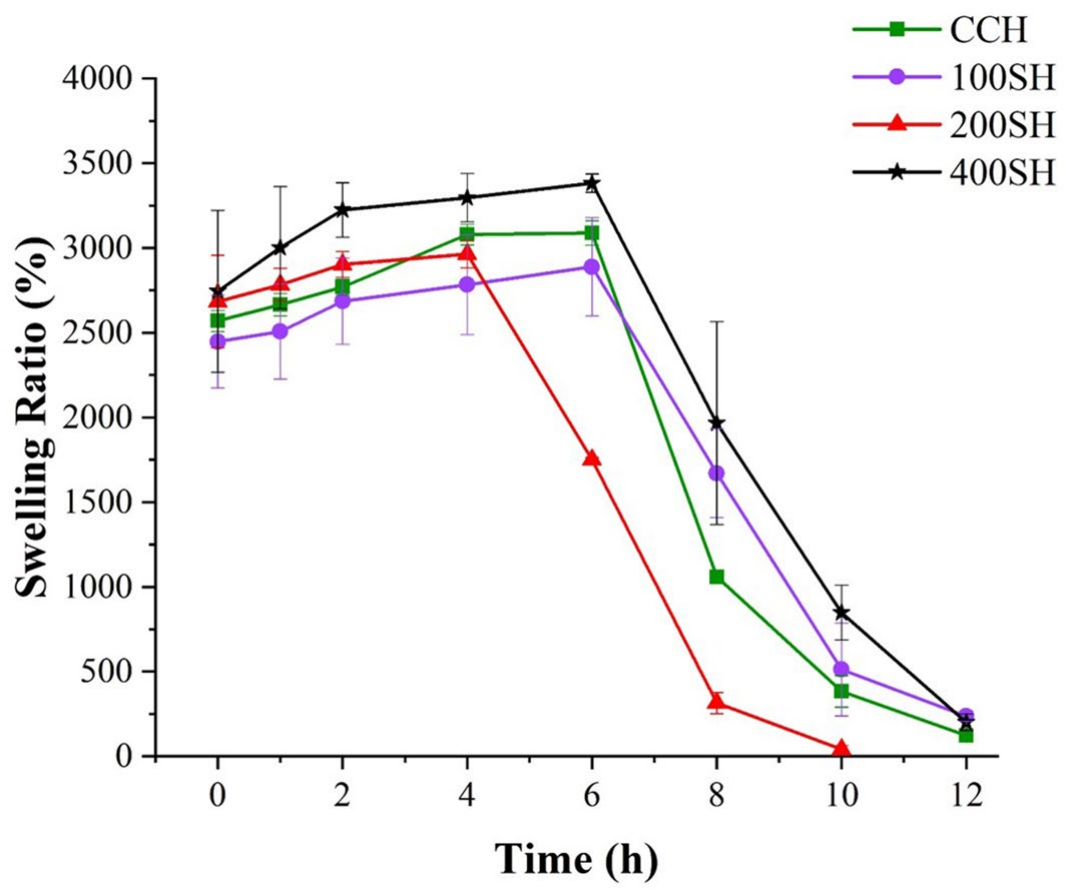
The Swelling ratio of hydrogels evaluated at a particular time from 0 to 12 h. Data are expressed as mean ± S.D (n = 3). CCH: κ-carrageenan oligosaccharides linked cellulose nanofibers hydrogel, 100SH: 100 mg surfactin and Herbmedotcin loaded hydrogel, 200SH: 200 mg surfactin and Herbmedotcin loaded hydrogel, and 400SH: 400 mg surfactin and Herbmedotcin loaded hydrogel.

The water absorption by hydrogels at different time intervals is given in Fig. 5. It was noted that hydrogels absorbed a large amount of water initially and later slightly increased with time. The maximum swelling ratio was observed up to 6 h. After, the swelling ratio was decreased suddenly in all hydrogels due to the breakdown of the dry hydrogel system in the presence of water. This may due to the brittle nature of the hydrogel. Literature mentioned that the bond-breaking was observed in highly swollen hydrogels was due to their brittle nature^41^.

### Antioxidant activity of hydrogels

**Figure 6:**
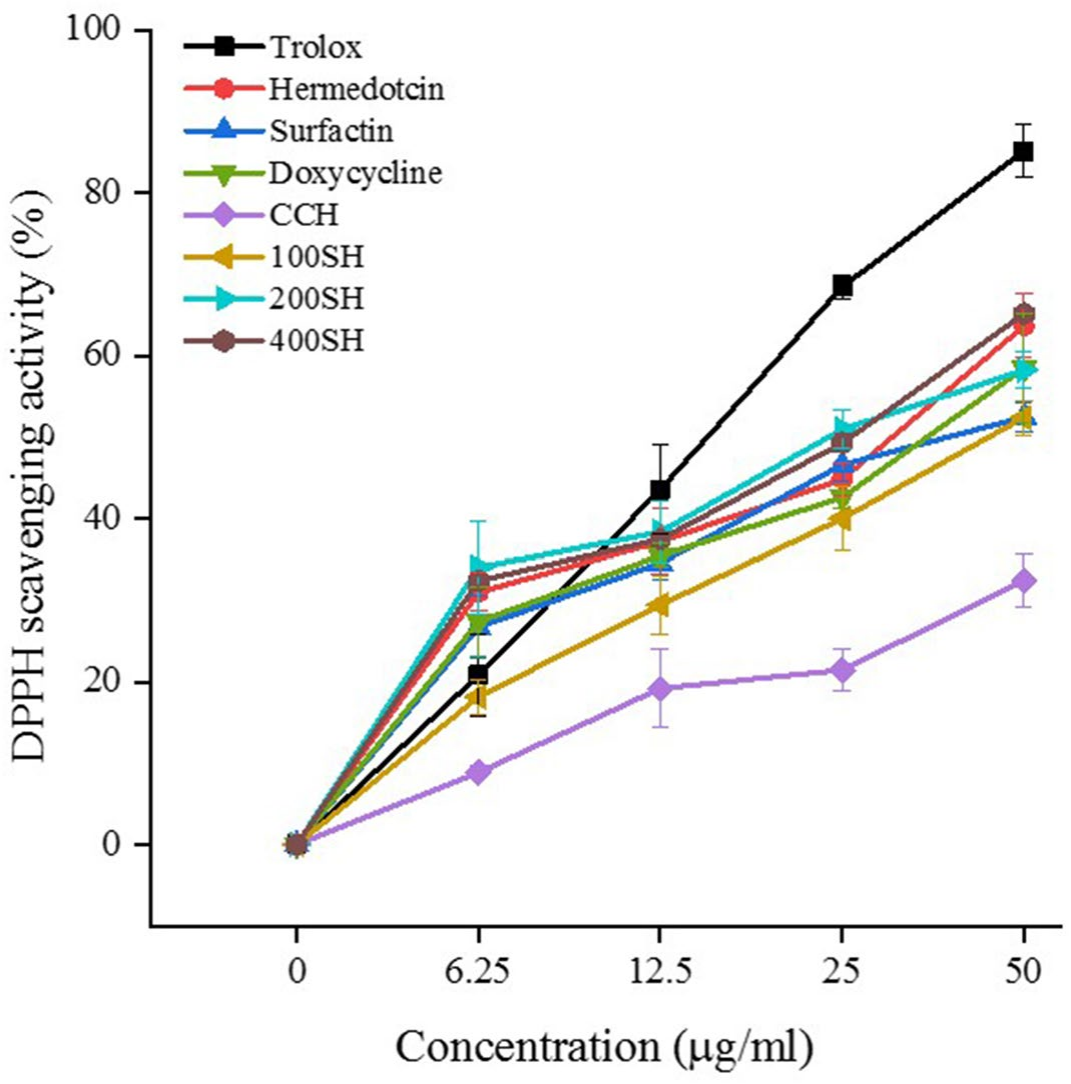
2,2-Diphenyl-1-picrylhydrazyl (DPPH) radical scavenging activity of surfactin, Herbmedotcin, doxycycline, CCH, 100SH, 200SH, and 400SH. Trolox was selected as standard. 100 µl of DPPH (0.004 g in 100 ml methanol) was mixed with an equal volume of sample for 30 min and the absorbance was measured at 517 nm. Data are expressed as mean ± S.D. (n = 3). CCH: ĸ-carrageenan oligosaccharides linked cellulose nanofibers hydrogel, 100SH: 100 mg surfactin and Herbmedotcin loaded hydrogel, 200SH: 200 mg surfactin and Herbmedotcin loaded hydrogel, and 400SH: 400 mg surfactin and Herbmedotcin loaded hydrogel.

The antioxidant activity of the samples was evaluated by the DPPH assay (Fig. 6). Trolox was taken as the standard. It was noted that more than 60% of scavenging activity was observed in the presence of Herbmedotcin and 400SH. The antioxidant activity of doxycycline was almost similar to 200SH and the activity of surfactin was slightly higher than 100SH. The maximum scavenging activity was observed in CCH was about 32%. It was assumed that the antioxidant activity of hydrogels may due to the synergetic effect of CO, surfactin, and Herbmedotcin.

### Well (ditch) plate method

**Figure 7:**
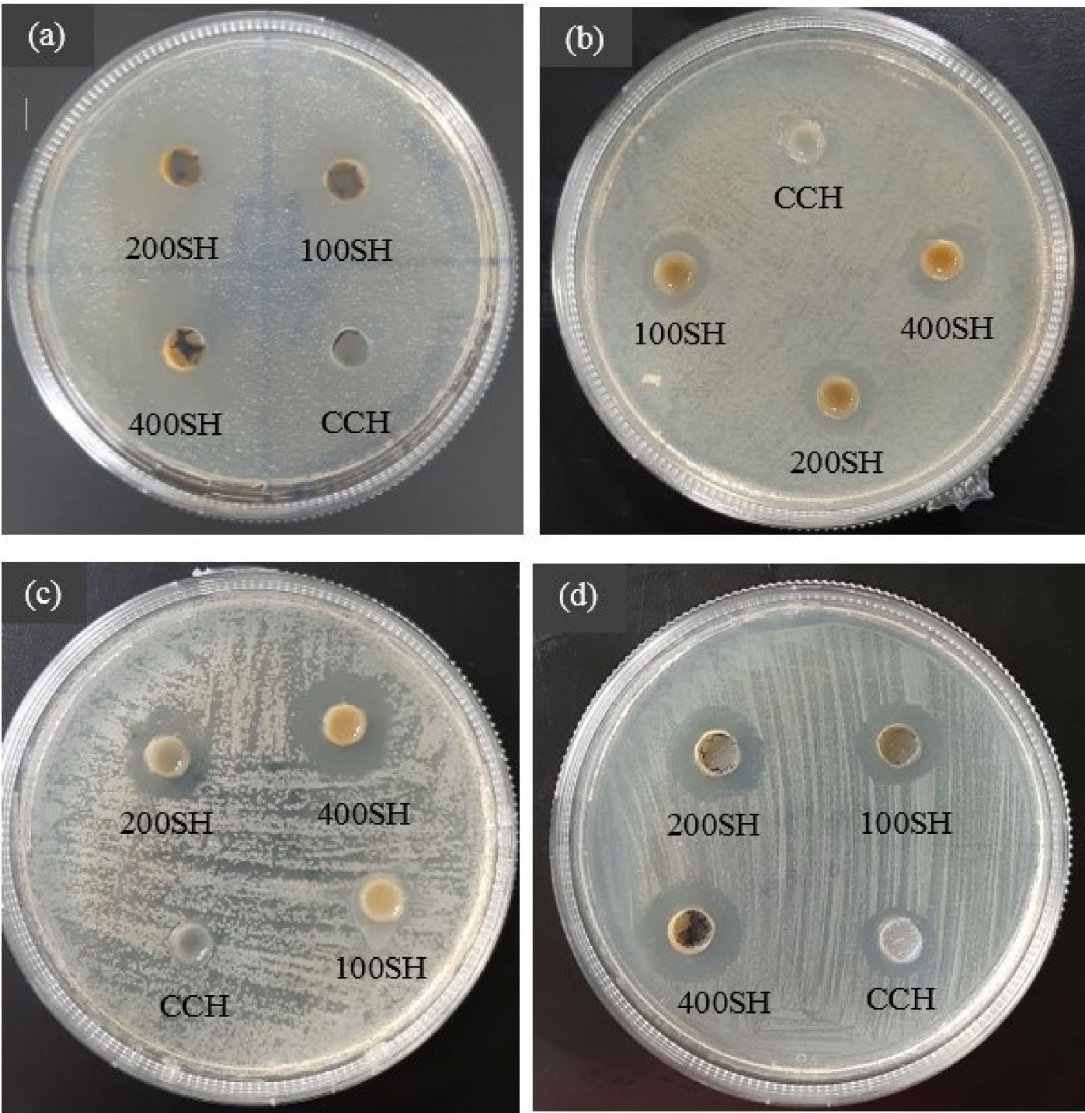
Antimicrobial activities of hydrogels against (**a**) *S. mutans*, (**b**) *P. gingivalis*, (**c**) *F. nucleatum*, and (**d**) *P. aeruginosa*. 100 µl of each sample was added to their respective wells made on a 10 µl bacteria (1 × 10^7^ CFU/ml) coated agar plate and the zone of inhibition was measured after 24 h. The experiment was performed in triplicates. CCH: κ-carrageenan oligosaccharides linked cellulose nanofibers hydrogel, 100SH: 100 mg surfactin and Herbmedotcin loaded hydrogel, 200SH: 200 mg surfactin and Herbmedotcin loaded hydrogel, and 400SH: 400 mg surfactin and Herbmedotcin loaded hydrogel.

Figure 7 and Table 1 showed the antimicrobial activity of hydrogels against four periodontal pathogens such as *S. mutans*, *P. gingivalis*, *F. nucleatum*, and *P. aeruginosa*. As shown in Fig. 7, drug-loaded hydrogels possessed antimicrobial activity against *S. mutans* in a concentration-dependent manner, at the same time the bacteria were resistant to CCH. The zone of inhibition (ZI) of doxycycline was slightly higher than 400SH whereas free Herbmedotcin and surfactin showed 11.66 mm and 13.33 mm ZI, respectively. This situation was almost similar to *P. gingivalis* but, *P. gingivalis* shown little bit sensitivity towards CCH. Only 400SH has higher ZI than Herbmedotcin and doxycycline. Hydrogels such as 200SH and 400SH showed antibacterial activity against *F. nucleatum* but the ZI was lesser than doxycycline*.*100SH and CCH did not show any activity against *F. nucleatum*. *P. aeruginosa* has shown sensitivity against all samples including CCH. The ZI of 200SH (18.00 mm) and 400SH (20.66 mm) was higher than doxycycline (17.84 mm). There were no major differences between the ZI of Herbmedotcin, surfactin, and CCH, in the case of *P. aeruginosa*.

**Table 1:** Zone of inhibition (ZI) of samples.

Microorganisms	Zone of inhibition (mm)						
	Doxycycline	Herbmedotcin	Surfactin	CCH	100SH	200SH	400SH
*Streptococcus mutans*	28.66 ± 2.51^a^	11.66 ± 1.52^d^	13.33 ± 1.52^c^	N	19.00 ± 2.00^b,c^	21.00 ± 1.73^b^	26.33 ± 1.52^a,b^
*Porphyromonas gingivalis*	25.00 ± 2.00^a^	16.33 ± 0.57^b^	12.66 ± 1.15^c^	0.9 ± 1.56^d^	11.66 ± 1.15^c^	14.33 ± 0.47^b,c^	18.33 ± 0.57^a,b^
*Fusobacterium nucleatum*	23.66 ± 1.64^a^	13.35 ± 3.54^c^	14.06 ± 2.92^c^	N	N	16.33 ± 2.34^b^	20.33 ± 0.63^a,b^
*Pseudomonas aeruginosa*	17.84 ± 1.38^a,b^	11.66 ± 0.53^c^	10.33 ± 2.73^c^	11.33 ± 3.64^b^	12.66 ± 0.28^b^	18.00 ± 2.34^a,b^	20.66 ± 1.25^a^

Data are expressed as mean ± S.D. (n = 3). Data are expressed as mean ± S.D. (n = 3). The values with different letters (a–d) represent a significant difference (p < 0.05) analyzed by one-way ANOVA followed by Tukey multiple comparison test.

CCH: κ-carrageenan oligosaccharides linked cellulose nanofibers hydrogel, 100SH: 100 mg surfactin and Herbmedotcin loaded hydrogel, 200SH: 200 mg surfactin and Herbmedotcin loaded hydrogel, and 400SH: 400 mg surfactin and Herbmedotcin loaded hydrogel.

### Minimum inhibitory concentration (MIC) and minimum bactericidal concentration (MBC) of hydrogels

The MIC and MBC of hydrogels against microorganisms were given in Table 2 and it was expressed as the percentage of its initial concentration (40 mg/ml). As shown in the table, CCH has not been shown any inhibitory effects on *S. mutans* but drug-loaded hydrogels inhibited the visible growth of *S. mutans* at particular concentrations. The MIC and MBC values of 100SH were the same (100%) while the MIC and MBC values of 200SH were 70% and 90%, respectively. 400SH inhibited the visible growth at 60% concentration and it prevents the growth at 70% concentration. In *P. gingivalis*, the MIC values of 100SH, 200SH, and 400SH were reduced in a concentration-dependent manner. The MBC values of 100SH and 200SH were the same (100%), while 80% were shown by 400SH. There was no MIC and MBC value for CCH in the case of *F. nucleatum*. The MIC value of 100SH was 100% but it does not possess any MBC value of up to 120%. At the same time, the MIC and MBC values of 400SH were 60%. It was understood that *P. aeruginosa* was sensitive to all the samples. The MIC and MBC value of CCH was 90% and 100%, respectively. The MIC values of 100SH and 200SH were the same (70%), but it was 40% for 400SH. 50% of 400SH inhibited the growth of *P. aeruginosa*, whereas 100SH and 200SH inhibited the growth at 90% and 70% concentration, respectively. From the table, the MBC value was 100% for 100SH (*S. mutans* and *P. gingivalis*) and 200SH (*P. gingivalis* and *F. nucleatum*) whereas the MBC value of 400SH was at 90% for *F. nucleatum*. So, 100% of concentrated samples were taken for further antimicrobial studies.

**Table 2:** Minimum inhibitory concentration (MIC) and minimum bactericidal concentration (MBC) of hydrogels.

Microorganisms	MIC (%)				MBC (%)			
	CCH	100SH	200SH	400SH	CCH	100SH	200SH	400SH
*Streptococcus mutans*	N	100	70	60	N	100	90	70
*Porphyromonas gingivalis*	100	80	70	50	N	100	100	80
*Fusobacterium nucleatum*	N	110	80	70	N	N	100	90
*Pseudomonas aeruginosa*	90	70	70	40	100	90	70	50

Data are expressed based on the percentage of initial concentration (40 mg/ml) of each drug (n = 3).

MIC: minimum inhibitory concentration, MBC: minimum bactericidal concentration, CCH: κ-carrageenan oligosaccharides linked cellulose nanofibers hydrogel, 100SH: 100 mg surfactin and Herbmedotcin loaded hydrogel, 200SH: 200 mg surfactin and Herbmedotcin loaded hydrogel, and 400SH: 400 mg surfactin and Herbmedotcin loaded hydrogel.

### Biofilm inhibition by crystal violet assay

**Figure 8:**
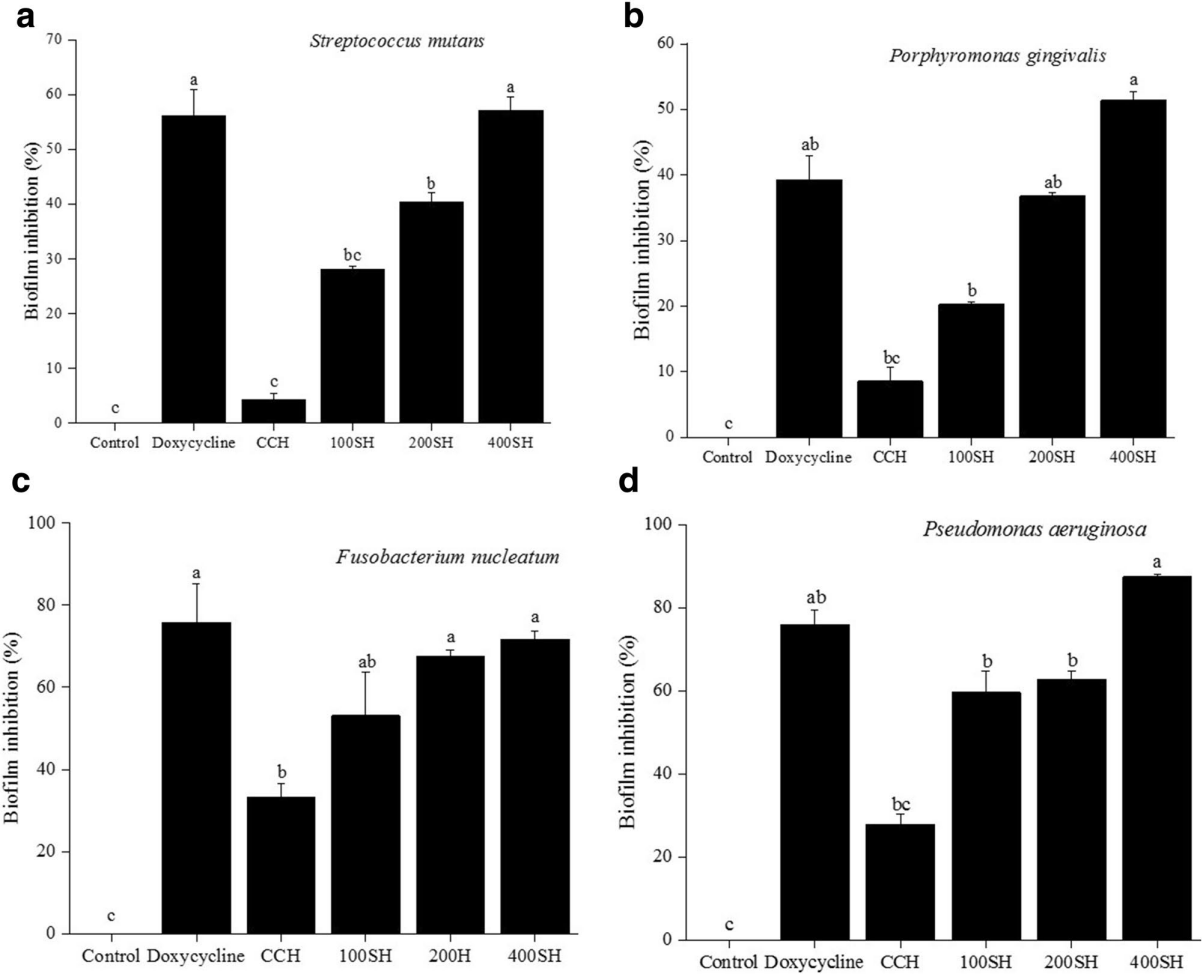
Quantification of biofilm inhibition of (**a**) *S. mutans*, (**b**) *P. gingivalis* (**c**) *F. nucleatum*, and (**d**) *P. aeruginosa*. The samples were stained with 0.1% crystal violet and the absorbance was measured at 595 nm. Data are expressed as mean ± S.D. (n = 3). The values with different letters (a–c) represent a significant difference (p < 0.05) analyzed by one-way ANOVA followed by Tukey multiple comparison test. CCH: κ-carrageenan oligosaccharides linked cellulose nanofibers hydrogel, 100SH: 100 mg surfactin and Herbmedotcin loaded hydrogel, 200SH: 200 mg surfactin and Herbmedotcin loaded hydrogel, and 400SH: 400 mg surfactin and Herbmedotcin loaded hydrogel.

Biofilm is the association of microbial species that are encased in a matrix^42^. The inhibition of biofilm by different samples towards periodontal pathogens were given in Fig. 8 and Supplementary Fig. S1. Doxycycline and 400SH shown more inhibitory effect on *S. mutans* and its activities were significantly higher than 100SH and 200SH. Like, in *P. gingivalis*, the inhibitory effect of 100SH, 200SH, and 400SH was increased. The highest percentage of biofilm inhibition was exhibited by 400SH and the next was doxycycline. There was no significant difference in the activity between doxycycline and 200SH. As compared to previous bacteria, the CCH treatment has some beneficial effects on *F. nucleatum*. There was no significant biofilm inhibition was observed between doxycycline, 200SH, and 400SH but it was higher than 100SH. The highest percentage of biofilm inhibition by 400SH on *P. aeruginosa* was observed and it was significantly higher than doxycycline. About 60% of biofilm inhibition was shown by 100SH and 200SH. CCH possessed more than 20% of inhibition activity in *P. aeruginosa* and *F. nucleatum* than *S. mutans* and *P. gingivalis*. Drug-loaded hydrogels produced biofilm inhibition after 24 h of treatment but 400SH has the highest biofilm inhibitory effect on all periodontal pathogens when compared to other hydrogels.

### Metabolic activity of bacteria

**Figure 9:**
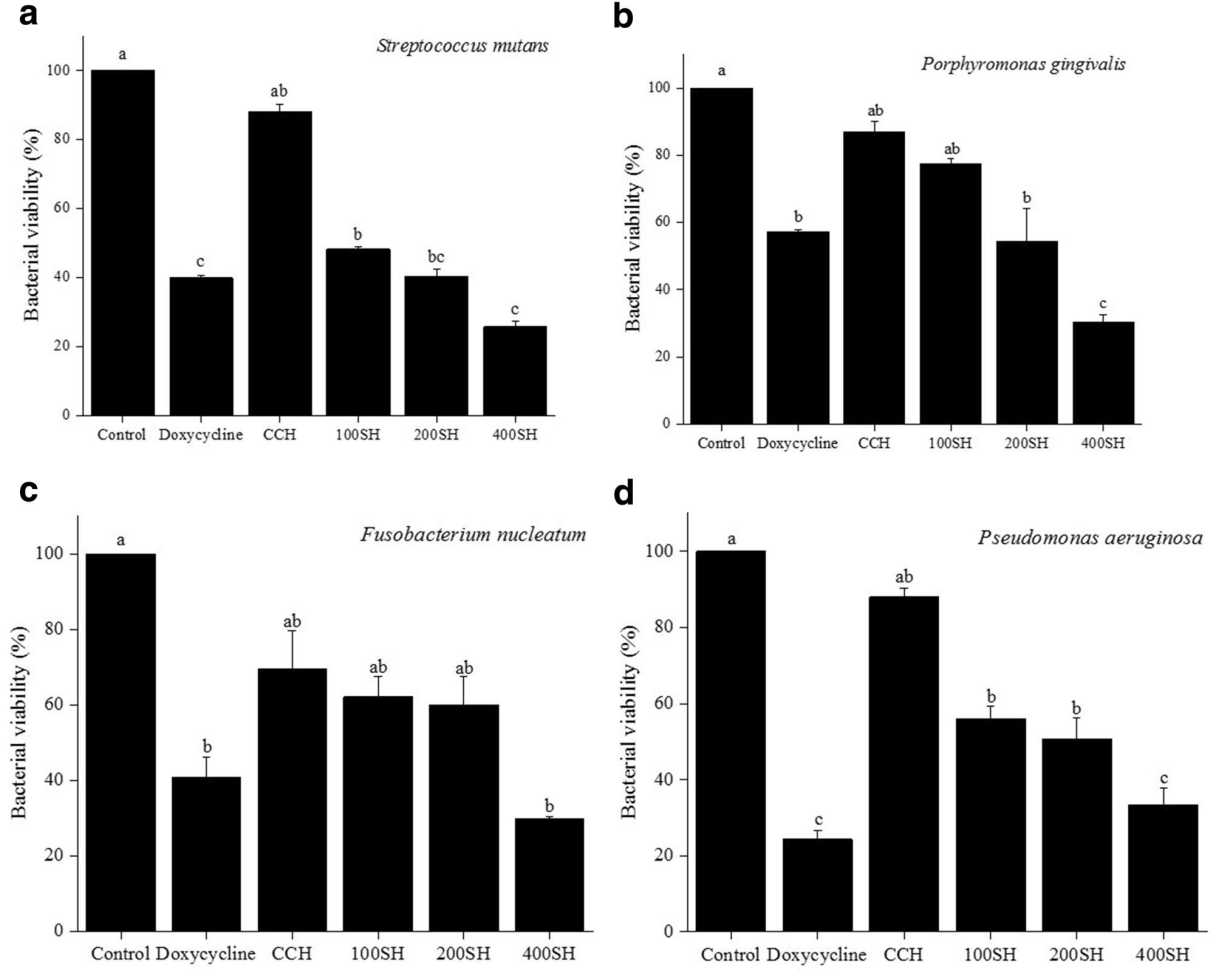
Evaluation of metabolic activities of (**a**) *S. mutans*, (**b**) *P. gingivalis*, (**c**) *F. nucleatum*, and (**d**) *P. aeruginosa*. Data are expressed as mean ± S.D. (n = 3). The samples were incubated with 100 µl of MTT and the absorbance was measured at 570 nm. The values with different letters (a–c) represent a significant difference (p < 0.05) analyzed by one-way ANOVA followed by Tukey multiple comparison test. CCH: κ-carrageenan oligosaccharides linked cellulose nanofibers hydrogel, 100SH: 100 mg surfactin and Herbmedotcin loaded hydrogel, 200SH: 200 mg surfactin and Herbmedotcin loaded hydrogel, and 400SH: 400 mg surfactin and Herbmedotcin loaded hydrogel.

The metabolic activity of the bacteria was estimated by MTT (3-(4,5-dimethylthiazol-2-yl)-2,5-diphenyltetrazolium bromide) assay (Fig. 9). It is the cheapest and simplest technique that helps to understand the physiological state of the bacteria^43^. The viability of *S. mutans* was highly reduced after treatment with doxycycline, 100SH, 200SH, and 400SH. 400SH showed the highest reduction viability and doxycycline came to the second position. As shown in the Fig. 9, the viability of *P. gingivalis* was dramatically reduced after 400SH treatment and the viability was reduced in an order of CCH > 100SH > 200SH > 400SH. There was no significant difference between the viability of doxycycline and 200SH groups and CCH and 100SH groups. 400SH did not possess any noteworthy difference in the viability of *F. nucleatum* when compared to doxycycline but it was much lesser than doxycycline. There were no significant variations in the metabolic activity of the *F. nucleatum* between CCH, 100SH, and 200SH groups. The lowest viability of *P. aeruginosa* was seen in the doxycycline-treated group and 400SH were come under the second position. 100SH and 200SH did not possess any significant difference in their activity towards *P. aeruginosa*.

### Malondialdehyde (MDA) assay

**Figure 10:**
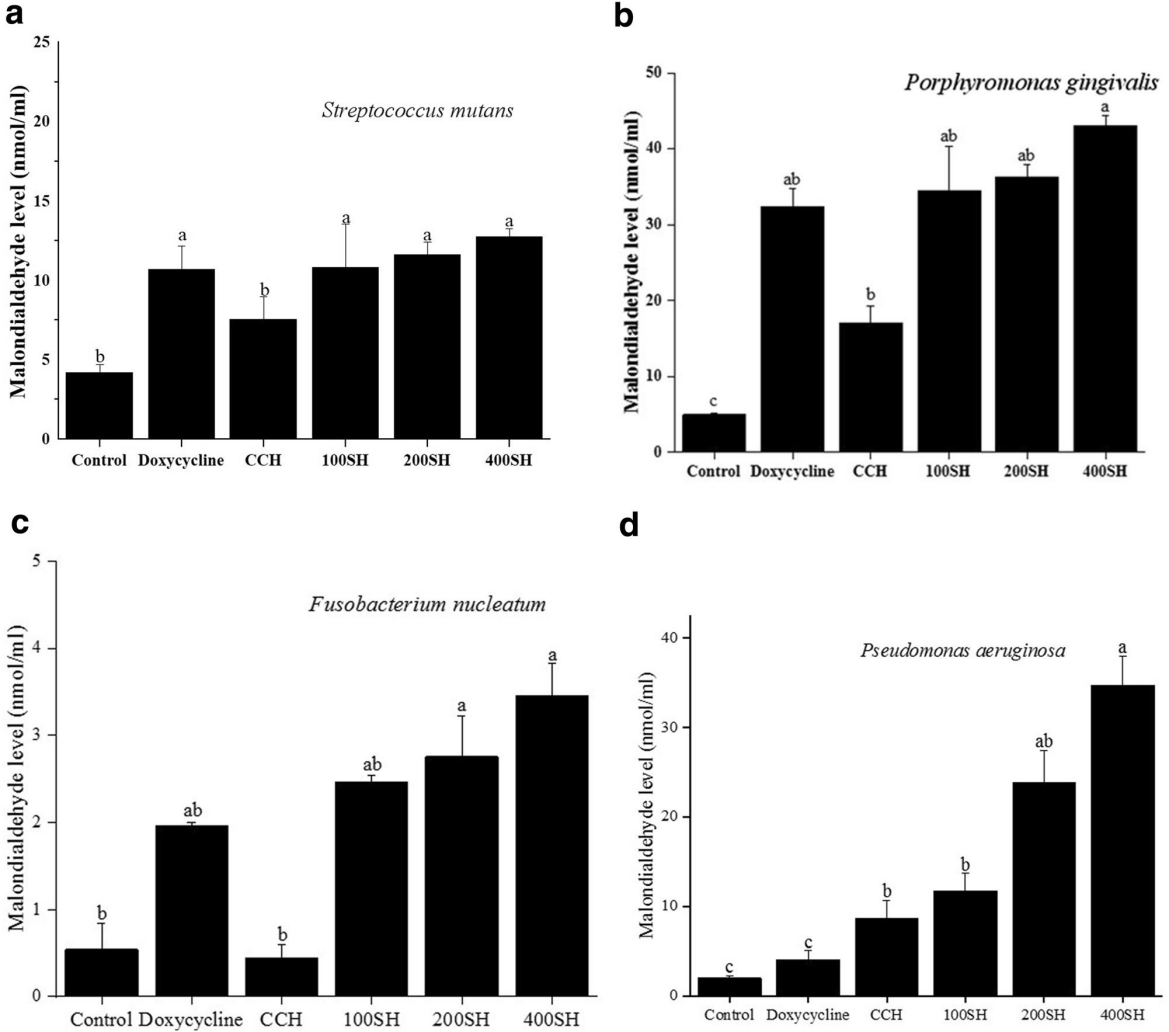
Malondialdehyde inhibition of (**a**) *S. mutans*, (**b**) *P. gingivalis*, (**c**) *F. nucleatum*, and (**d**) *P. aeruginosa* by hydrogels. The samples were treated with 200 µl MDA and the absorbance was measured at 532 nm. Data are expressed as mean ± S.D. (n = 3). The values with different letters (a–c) represent a significant difference (p < 0.05) analyzed by one-way ANOVA followed by Tukey multiple comparison test. CCH: κ-carrageenan oligosaccharides linked cellulose nanofibers hydrogel, 100SH: 100 mg surfactin and Herbmedotcin loaded hydrogel, 200SH: 200 mg surfactin and Herbmedotcin loaded hydrogel, and 400SH: 400 mg surfactin and Herbmedotcin loaded hydrogel.

As shown in Fig. 10, the MDA level was highly reduced in the control group for all bacteria. In *S. mutans*, the highest level of MDA was observed in 400SH but it was not significantly different from other groups such as doxycycline, 100SH, and 200SH. This situation was slightly similar in the case of *P. gingivalis*, in which the highest MDA level was observed in 400SH. Doxycycline, 100SH, and 200SH have no significant difference in the MDA level but it was higher than CCH. In *F. nucleatum*, a low level of MDA was observed in positive control when compared to 100SH, 200SH, and 400SH. The highest MDA level was seen in the 400SH group but it was not significantly differed from 200SH. As shown in the Fig. 10, the level of MDA was increased in the CCH group than doxycycline in the case of *P. aeruginosa*. The highest MDA level was observed in 400SH followed by 200SH.

### Acridine orange (AO) assay

**Figure 11:**
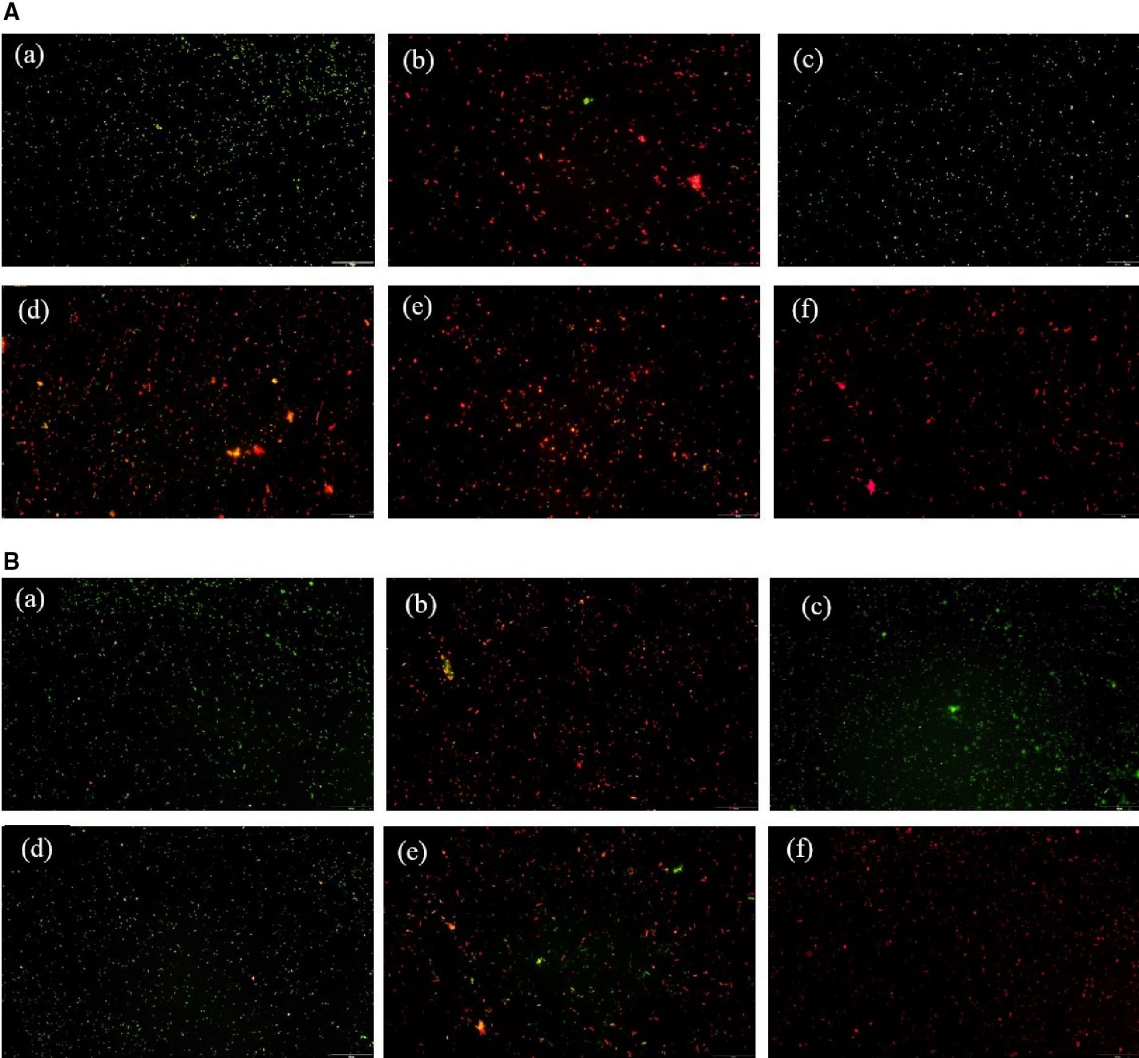

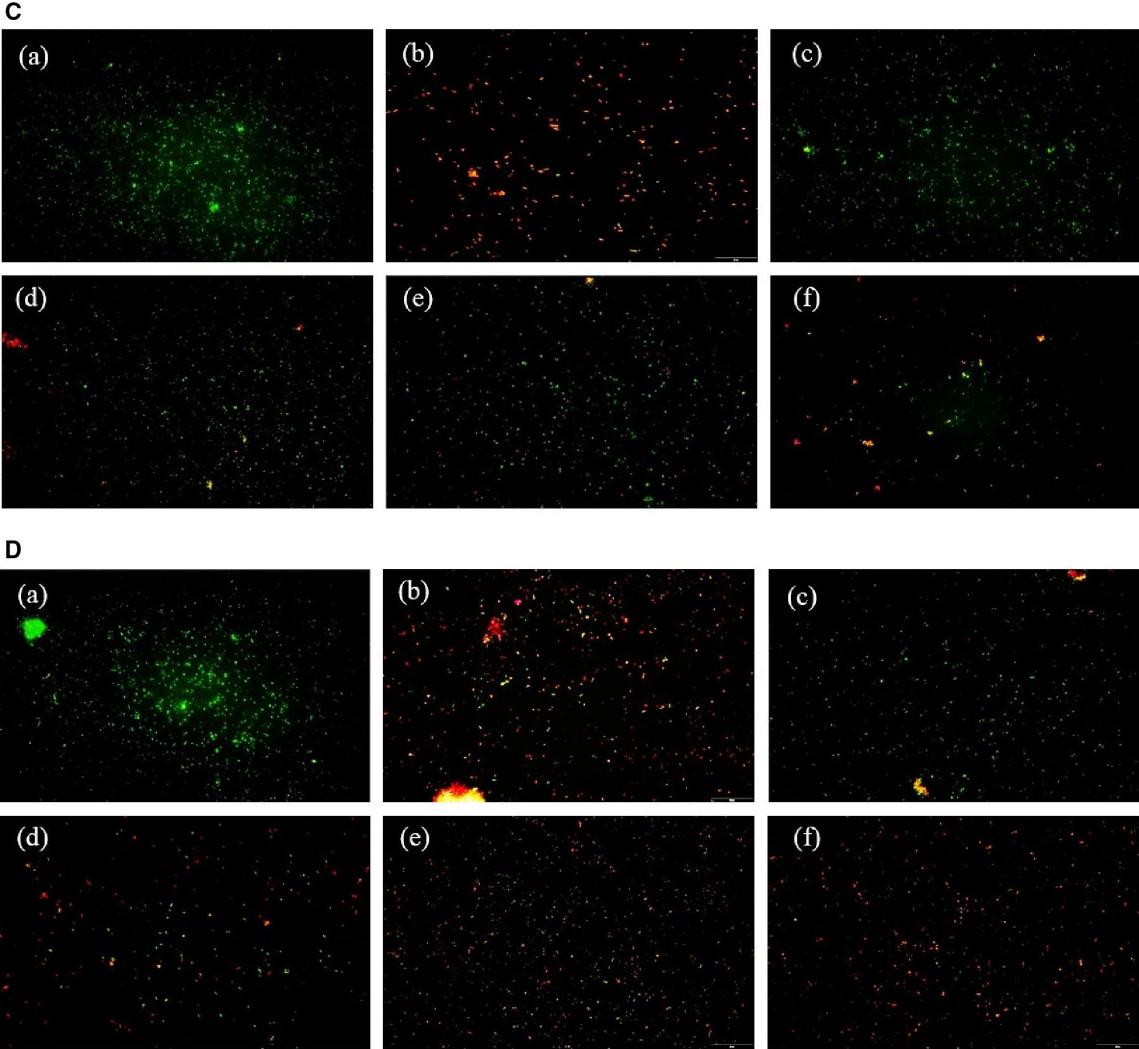
Acridine orange assay of (**A**) *S. mutans*, (**B**) *P. gingivalis*, (**C**) *F. nucleatum*, and (**D**) *P. aeruginosa*. The scale bar represents 20 µm. (a) Control; (b) doxycycline; (c) CCH; (d) 100SH, (e) 200SH, (f) 400SH. CCH: κ-carrageenan oligosaccharides linked cellulose nanofibers hydrogel, 100SH: 100 mg surfactin and Herbmedotcin loaded hydrogel, 200SH: 200 mg surfactin and Herbmedotcin loaded hydrogel, and 400SH: 400 mg surfactin and Herbmedotcin loaded hydrogel.

The bacteria were treated with samples for 24 h and then stained with AO. Images of AO assay was elucidated in Fig. 11. It was observed that *S. mutans* were highly viable in the control and CCH group. Very few numbers of viable cells were seen in doxycycline, 100SH, and 200SH whereas 400SH almost promoted 100% bacterial death. However, the situation was different in *P. gingivalis*. Along with control and CCH groups, 100SH also showed more viable cells and 200SH has an approximately higher number of viable cells as compared to doxycycline and 400SH. The viability of the *F. nucleatum* was extremely increased in all groups except doxycycline. Like *S. mutans*, viable *P. aeruginosa* was highly observed in control and CCH groups. 100SH showed an almost equal number of viable and non-viable cells whereas the number of dead cells more seen in doxycycline 200SH, and 400SH. It was confirmed that these hydrogels can kill 4 kinds of bacteria in its higher form (200SH and 400SH) together with doxycycline.

### Cytotoxicity of hydrogels in human gingival fibroblast (HGF) cells

**Figure 12:**
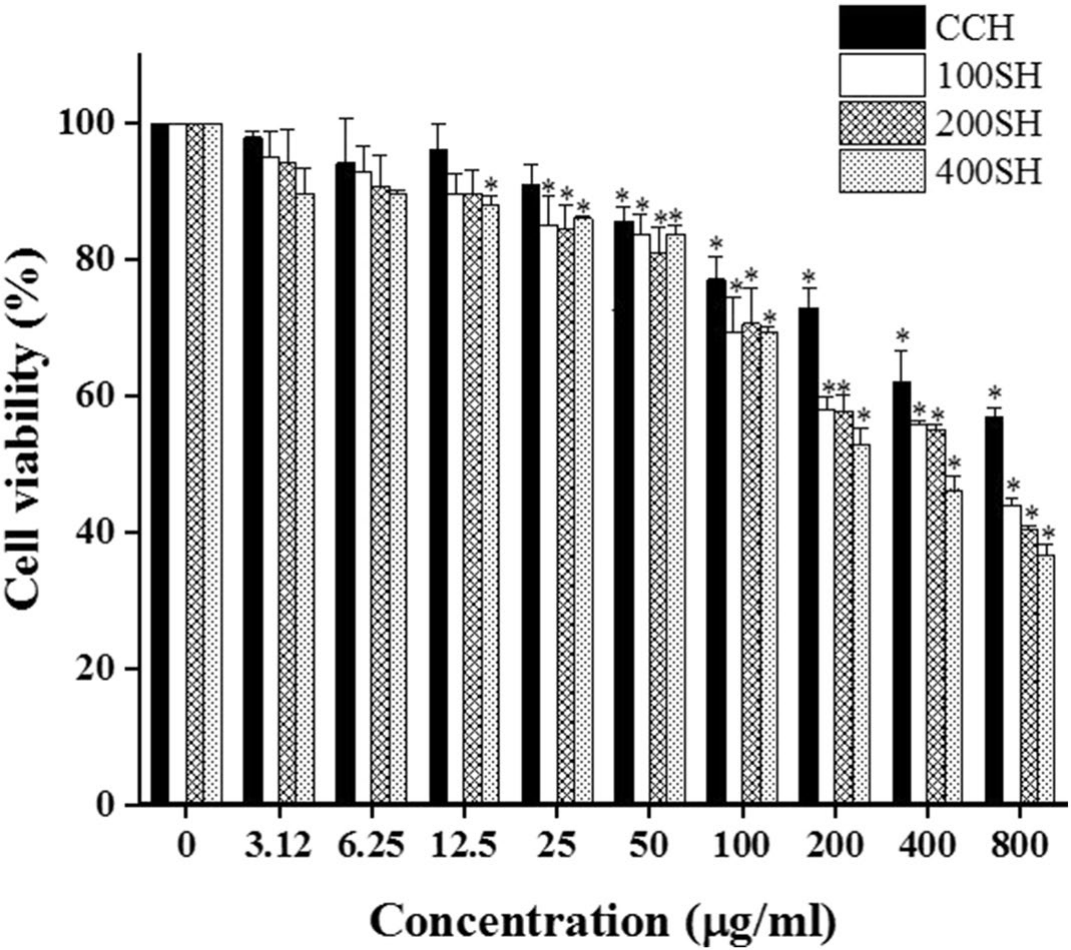
Cell viability of HGF cells (1 × 10^4^ cells/well) in the presence of hydrogels after 24 h analyzed by MTT assay. Data are expressed as mean ± S.D. (n = 3). The asterisk (*) indicated the significant differences of the groups from control at p < 0.05 analyzed by one-way ANOVA followed by Tukey multiple comparison test. CCH: κ-carrageenan oligosaccharides linked cellulose nanofibers hydrogel, 100SH: 100 mg surfactin and Herbmedotcin loaded hydrogel, 200SH: 200 mg surfactin and Herbmedotcin loaded hydrogel, and 400SH: 400 mg surfactin and Herbmedotcin loaded hydrogel.

MTT assay was used to determine the viability of the cells. The cell viability is linearly proportional to its mitochondrial activity^44^. Figure 12 showed the viability of the HGF cell line after treated with different samples. It was noted that the cell viability was decreased with an increase in the concentration of each sample. As compared to other groups, CCH showed higher viability. It was also noted that the cell viability of all groups significantly differed from the control group from 50 µg/ml concentration. The cell viability of all samples was dropped below 80% from 100 µg/ml concentration. There was no big difference in cell viability between 100SH, 200SH, and 400SH but the viability was slightly reduced in the 400SH group when compared to others. All samples-treated cells exhibited more than 80% cell viability up to 50 µg/ml concentration. So, this concentration was selected for performing further analysis.

### Oxidative stress

**Figure 13:**
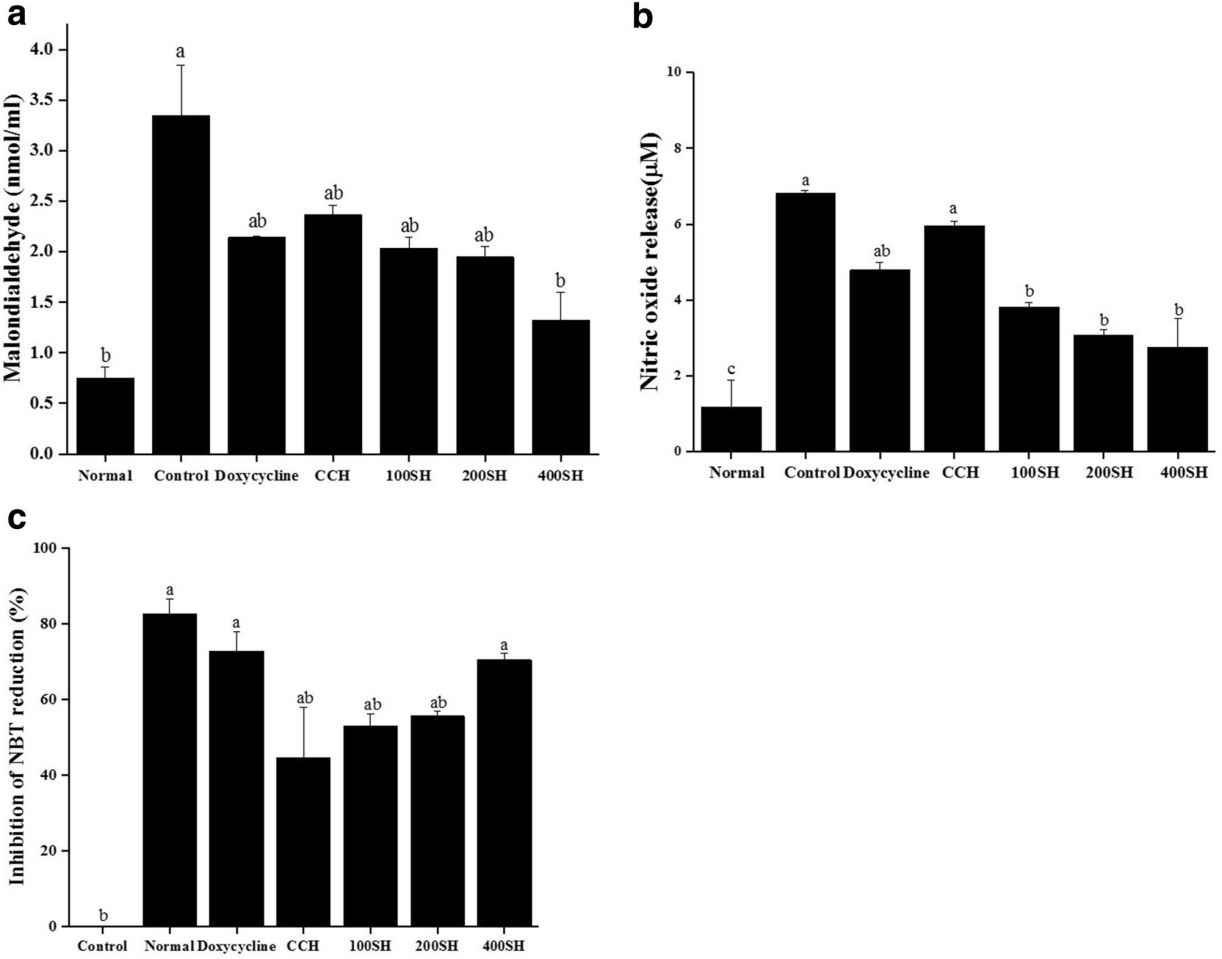
The (**a**) MDA level, (**b**) nitric oxide production, and (**c**) inhibition of NBT reduction in hydrogel treated LPS stimulated cells. The cells (1 × 10^4^ cells/well) were pre-treated with 20 µl of samples (50 µg/ml) and 10 µl of LPS (1 µg/ml). Treated cells were incubated with 200 µl MDA reagent and the absorbance was measured at 532 nm to evaluate the MDA production. For NO oxide production determination, 50 µl of cell supernatant was mixed with 50 µl Griess reagent. The absorbance was measured at 540 nm after 10 min of incubation. 300 µl of NBT solution was mixed with treated cells and the absorbance was measured at 570 nm to determine NBT reduction. Data are expressed as mean ± S.D. (n = 3). The letters (a–c) indicated the significant differences of the groups at p < 0.05 analyzed by one-way ANOVA followed by the Tukey multiple comparison test. CCH: κ-carrageenan oligosaccharides linked cellulose nanofibers hydrogel, 100SH: 100 mg surfactin and Herbmedotcin loaded hydrogel, 200SH: 200 mg surfactin and Herbmedotcin loaded hydrogel, and 400SH: 400 mg surfactin and Herbmedotcin loaded hydrogel. Normal: Cells without any treatment; Control: Cells were treated with only lipopolysaccharide.

An inflammatory condition in HGF cells was evaluated by performing MDA, NO, and NBT analysis (Fig. 13). MDA production was evaluated as a marker to determine the oxidative stress in LPS stimulated HGF cells. The cells were subjected to samples treatment after LPS induction. The MDA level was higher in the control group and lower in the normal group. There was no significant difference in the MDA level between doxycycline, CCH, 100SH, and 200SH but it was significantly higher than 400SH. So, it was confirmed that the hydrogels were able to reduce the MDA level under inflammatory conditions. Excessive production of ROS is a sign of oxidative stress and it causes damage to DNA, protein, and lipids^45^. Previous literature indicated that the systemic inflammatory responses can be evaluated from NO production^46^. It was observed that nitric oxide production was highly increased in the control group. As compared to doxycycline, drug-loaded hydrogel groups showed better results in terms of NO production. There was no significant difference between 100SH, 200SH, and 400SH but lower NO generation was observed in 400SH when compared to others. The ROS production in hydrogels treated inflammatory cells were again confirmed by NBT assay. It is used to determine the production of superoxide anion (O_2_^−^) by cell^47^. A high reduction of superoxide anion production was observed in normal, doxycycline, and 400SH treated groups. CCH, 100SH, and 200SH also reduced the production of superoxide anion when compared to the control group. So, it was understood that hydrogels can reduce the production of ROS, and thereby it can protect the cells from oxidative stress.

### Cytokines production

**Figure 14:**
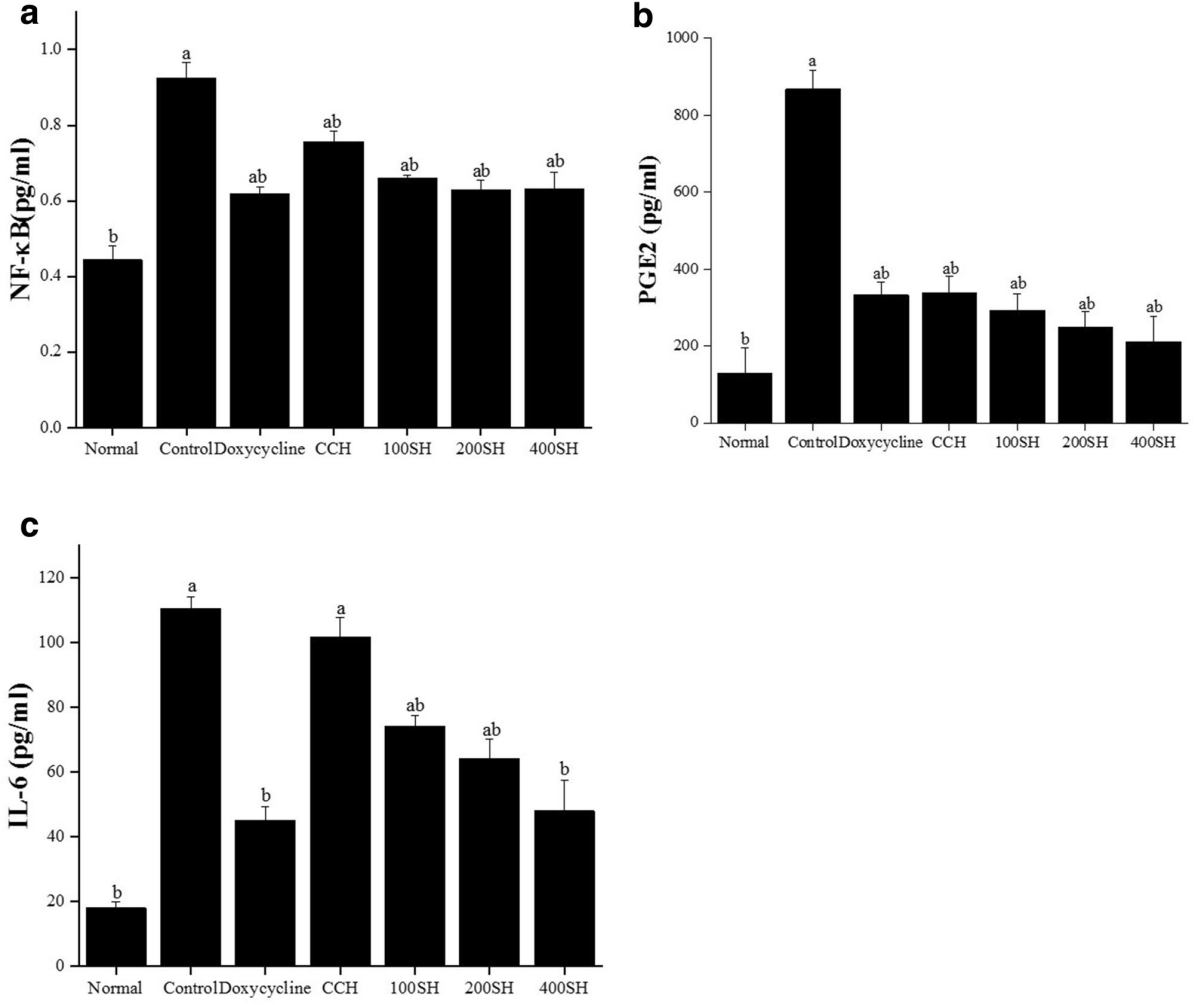
Level of (**a**) NF-κB, (**b**) PGE2, and (**c**) IL-6 in hydrogel treated LPS-stimulated cells. The cells (1 × 10^4^ cells/well) were pre-treated with 20 µl of samples (50 µg/ml) and 10 µl of LPS (1 µg/ml). All experiments were performed according to the manufacturer’s protocol. Data are expressed as mean ± S.D. (n = 3). The letters (a–b) indicated the significant differences of the groups at p < 0.05 analyzed by one-way ANOVA followed by the Tukey multiple comparison test. CCH: κ-carrageenan oligosaccharides linked cellulose nanofibers hydrogel, 100SH: 100 mg surfactin and Herbmedotcin loaded hydrogel, 200SH: 200 mg surfactin and Herbmedotcin loaded hydrogel, and 400SH: 400 mg surfactin and Herbmedotcin loaded hydrogel. Normal: Cells without any treatment; Control: Cells were treated with only lipopolysaccharide.

Literature showed that in cultural periodontal cells, the activation of NF-κB by IL-1β and TNF-α was observed and this may lead to the production of prostaglandin and matrix metalloproteinase (MMP)^48^. The production of NF-κB, PGE2, and IL-6 after treatment were given in Fig. 14. The production of NF-κB was higher in the control group and lower in the normal group. It was noted that there was no significant difference between doxycycline and hydrogels but CCH has higher NF-κB expression than others. The level of PGE2 and IL-6 was also higher in the control group and it was significantly lower in the normal group. Like NF-κB, the expression PGE2 was not significantly differed between other groups, and comparatively lower production was seen in 400SH. But this situation was quite different in IL-6. CCH group also showed a higher level of IL-6 like the control group and the level was lowered in the following order CCH > 100SH > 200SH > 400SH > doxycycline. 400SH and doxycycline have reduced IL-6 production below 60 pg/ml and it was significantly lower than 100SH and 200SH.

## Discussion

Periodontitis is an inflammatory disease primarily resulted from the microbial accumulation and it is estimated that about 47.2% of the U.S. adult population was affected by periodontitis^49^. Periodontitis is developed due to the formation of biofilms composed by enzymes, protein epithelial cells, and food residues and the teeth provide a suitable surface for bacterial attachment and progression^50^. Literature mentioned that there are five different ecological niches such as teeth, saliva, periodontal pockets, gingival sacculus, and tongue existed for the initiation and progression of periodontitis^51^. The current study is focusing on four major bacteria such as *P. gingivalis*, *S. mutans*, *F. nucleatum*, and *P. aeruginosa,* which cause periodontitis directly or indirectly. *P. gingivalis* is a gram-negative anaerobic bacterium that initiates the inflammation by invading the epithelial cells. Besides, the major component of gingival connective tissue called gingival fibroblast may directly interact with *P. gingivalis* and its bacterial products called LPS^52^. *S. mutans* is another bacterium that indirectly leads to the progression of periodontitis. It is known as the major etiological agent for dental caries. The dental caries is formed due to the sensitivity of the teeth towards acidic pH and resulted in the dissolution of enamel. The literature mentioned that both dental caries and periodontitis linked directly or indirectly due to some common contributory factors and the pockets formed as a result of dental caries may facilitate the progression of periodontitis^53^. Like *P. gingivalis*, *F. nucleatum* is also an anaerobic gram-negative bacterium responsible for the progression of periodontitis. It increases pocket depth, inflammation, and plaque formation. It encodes several adhesins and binds to several mammalian cells. The virulent mechanism of *F. nucleatum* has been classified into the induction of host immune response, colonization, and dissemination^54^. A previous study reported the high prevalence of *P. aeruginosa* in the subgingival biofilm under different periodontal conditions and they isolated Pseudomonas species from dental biofilms and mucosa^55^. However, it is commonly associated with the generation of pulmonary disease. However, literature stated that the oral cavity is known as the important reservoir for *P. aeruginosa*. So, the eradication of *P. aeruginosa* is also crucial to maintaining normal health. Due to the host-bacterial interaction, an acute inflammatory response will occur and neutrophils invade the connective tissue and later interact with other immune cells. As a result, cytokines such as IL-6, IL-17, TNF-α, IL-1α, and IL-1β are produced^56^. IL-17 promotes the production of chemokines, matrix metalloproteinases (MMPs), osteoblast expression of receptor activator of nuclear factor-kB ligand (RANKL), and other tissue deductive molecules^57^. Recent literature mentioned that LPS can act as a mediator for inflammation in the gingival area by enhancing the production of cytokines such as IL-6, TNF-α, and IL-1β. In addition to this, ROS overproduction and the activity of certain cells such as neutrophils, leucocytes, T lymphocytes, and plasma cells may also attribute to LPS-induced periodontitis^58^. Also, receptors such as TLR-4 and TLR-2 are connected to the pathology of periodontitis. The activation of these receptors will lead to the activation of T helper cell 2 and subsequent production of pro-inflammatory cytokines^59^.

The current study is focusing on the in vitro antibacterial and anti-inflammatory properties of surfactin and Herbmedotcin-loaded CO-CNF hydrogels. A local drug delivery system provides the attachment and preventing the side effects of free-drugs. The main aim of the drug delivery system is to deliver antimicrobial agents in those areas where mechanical scaling instruments cannot access. Besides, this material should have cytocompatibility and biocompatibility. The hydrogels were prepared from a composite nanoparticle (CO-CNF) composed of CNF and CO^15^. CNF is widely used for hydrogel preparation due to its flexibility and entanglement formation^14^. Lohrasbi et al. identified that the addition CNF improves the gelation time and stability of collagen hydrogels^60^. Like cellulose, CO is also a natural polymer and it mimics the glycosaminoglycan structure^61^. So, the combined use of CNF and CO provides a suitable platform for drug delivery. Herbmedotcin is a positively charged, safe, and effective antibacterial agent widely used in healthcare, agriculture, and aquaculture^20,62^. Studies were done by Park et al. supported the usage of surfactin as a candidate to prevent inflammatory disease, especially periodontitis^63^.

Recent literature mentioned that nanostructured hydrogels can facilitate the biological functions and tissue growth by stimulating interactions between cells and their environment^64^. The morphology of the hydrogels was evaluated by SEM analysis (Fig. 1). This technique helps to understand the porosity and nature of the hydrogels^65^. Because of the highly reactive carbon–carbon double bonds, MBAA has been used as a cross-linking agent and it can form a three-dimensional network structure by interacting with certain groups such as –NH_2_, –OH, and –COOH^66^. The smooth surface observed in the hydrogels was due to the cross-linking nanofibers and the blocks were obtained due to the cross-linking of CO. Previous study also reported the smooth surface nature of freeze‐dried NFC hydrogel in presence of calcium dinitrate (CaN_2_O_6_)^67^.

The FTIR analysis confirms the interactions between the CO–CNF, surfactin, and Herbmedotcin (Fig. 2). Moreover, this material has high thermal stability (Fig. 3) and it might be due to the strong bonding between the polymers and drugs^68^. In addition to high thermal stability, the high swelling ratio was also observed in hydrogels. The previous study stated that the network structure of the hydrogels are formed by either physical interaction or chemical interaction and the integrity and swelling of the hydrogels are depending upon the degree of cross-linking^69^. As shown in Fig. 5, the swelling ratio was increased suddenly and continued up to 6 h. After, it was dropped due to the breakdown of the hydrogel system. The collapse of the hydrogel system may result from the brittleness nature of the dried hydrogel system. Previous study mentioned the brittleness nature of highly cross-linked hydrogels^69^. The swelling ability of hydrogels was due to the presence of hydrophilic groups present in the CNF and CO. The high swelling ability of nanocellulose hydrogel is due to its osmotic effects because the ions present in the external solution are diffused to the center of the gel. As a result, the presence of counterions around the charged polymers in aqueous media causes electrostatic neutrality^70^. Hezaveh and Muhamad reported the hydrogen bond breaking and subsequent diffusion of water in κ-carrageenan and hydroxyethyl cellulose-based hydrogels^71^.

It was known that excess production of ROS leads to the destruction of periodontal tissues by lipid peroxidation, release of cytokines, protein damage, DNA damage, and oxidation of important enzymes^72^. DPPH radical scavenging assay was conducted to evaluate the antioxidant activity of the hydrogels (Fig. 6). The mechanism behind the DPPH assay is based on the antioxidant ability of the samples to quench the DPPH and the dark purple color of the DPPH is changed to colorless^73^. It was understood that 400SH and Herbmedotcin have high scavenging activity than others. It may be due to the synergetic effect of Herbmedotcin, surfactin, and CO. Literature stated that the DPPH radical scavenging activity of surfactin is due to the presence of peptide ring and fatty acid chain^74^. Herbmedotcin has also a major role in antioxidant activities because it contains effective pure compounds from herbs^20^. The ROS scavenging activity of low molecular weight κ-carrageenan is closely related to the degree and position of sulfation, composition of monosaccharides, and molecular weight^75,76^.

Periodontitis is an inflammatory disease associated with biofilm formation by the dysbiotic bacterial community. Antimicrobial activity of hydrogels was primarily evaluated by agar ditch plate method against four periodontal pathogens such as *P. gingivalis*, *S. mutans*, *F. nucleatum*, and *P. aeruginosa* (Fig. 7). It was observed that *P. gingivalis* and *P. aeruginosa* were a little bit sensitive to CCH and better zone of inhibition of 200SH and 400SH may due to the high concentration of the antibacterial agents. Literature mentioned that MIC value is not accurate to determine whether the antibiotic is bacteriostatic or bactericidal. However, MBC value helps to choose the lowest concentration of antibiotics that kill (99.9%) the bacteria^77^. According to our study (Table 2), the MBC values of hydrogels were ranges from 50 to 100%. The MBC values of 100SH were 100% for two bacteria, 90% for another one. It was also similar to 200SH, except there were 90% and 70% MBC values for *S. mutans* and *P. aeruginosa*, respectively. The situation was different in the case of 400SH, in which the MBC values were ranging from 80 to 50%. Thus, to maintain the constituency, 100% concentration was selected for further studies while considering all bacteria. The antimicrobial activity of hydrogels was potentially due to the synergetic effect of all components present in the hydrogel. It was also noted that the activities of hydrogels were varied according to each bacterium. The previous study reported that the antimicrobial activity of surfactin involves the disruption of bacterial membrane followed by the leakage of the cellular component while Herbmedotcin uses its positive charge to attach the lipid membrane and cause bacterial lysis^20,78^. It was also noted that the antibacterial activity of oxidized form of κ-carrageenan was reported against *P. aeruginosa*. Literature mentioned that κ-carrageenan causes bacterial death by damaging the bacterial cell wall and cytoplasmic membrane^79^.

During the adverse condition, different types of bacteria can colonize in the oral cavity and facilitate various interactions to initiate biofilm formation. Streptococcus and Actinomyces species lay the foundation for the development of bacterial biofilm^80^. Later, species such as Fusobacterium and Veillonella, etc. aggregate to colonizers and finally, it organized to form a complex matter formed by Corynebacterium, Streptococcus, Porphyromonas, Haemophilus/Aggregatibacter, Neisseriaceae, Fusobacterium, Leptotrichia, Capnocytophaga, and Actinomyces^80^. The Coaggregation of these species is happening due to intergeneric specific cell-to-cell recognition via surface adhesins and receptors^81^. The inhibition of biofilm by hydrogels were given in Fig. 8. It was noted that 400SH shown higher percentages of biofilm inhibition similar to doxycycline. Lowest biofilm inhibition (50%) by 400SH was observed in *P. gingivalis* but more than 80% of biofilm inhibition was seen in *P. aeruginosa*.

The production of ROS was drastically increased due to the innate immune system under inflammatory conditions and leads to oxidative stress and cell damage^82^. During phagocytosis, neutrophils produce O_2_^−^ by respiratory burst and these O_2_^−^ are converted to singlet oxygen (^1^O_2_), hypochlorous acid (HOCl), hydrogen peroxide (H_2_O_2_), and hydroxyl radical (OH·)^82^. It will cause various adverse damages, specifically lipid peroxidation. MDA is known as the end product of lipid peroxidation. Figure 10 showed that hydrogels were able to increase MDA production in four bacteria, especially by 200SH and 400SH. It was also understood that the metabolic activity of each bacteria was dropped after the treatment with hydrogels. This may reveal that the hydrogels cause oxidative stress in bacteria and leads to the reduction of their metabolic activity and finally resulted in cell death (Figs. 9, 11).

The safety of the proposed drug is mostly evaluated based on cell viability and cytotoxicity assays. A cytotoxic drug is defined as a compound that affects cell growth, interferes with cellular attachment, causes morphological changes, and finally reduce overall viability^83^. HGF cells were selected to evaluate the anti-inflammatory properties of the hydrogels. It is known as the most abundant cells in the gingival connective tissue and expresses cell surface CD14, TLR4, and MD-2 and produce pro-inflammatory cytokines, such as IL-6 and IL-8, upon LPS stimulation^84^. MTT assay is a colorimetric assay widely used for determining the viability of cells. The principle behind this assay is based on the dehydrogenases present in the mitochondria of viable cells and it converts the tetrazolium compound into formazan crystals^85^. The viability of HGF cells in the presence of each hydrogel was given in Fig. 12. It was noted that up to 50 µg/ml concentration, all the hydrogels maintained more than 80% of cell viability. So, this concentration was selected for further studies. It was known that the major mechanism behind periodontitis is the oxidative stress-mediated inflammatory pathway. The interaction between the bacterial biofilms and resident cells are leading to the inflammatory response. The bacterial outer membrane is consists of LPS and it can trigger the release of cytokines such as IL-6, IL-1β, and TNF-α^86,87^. In addition to this, excessive production of ROS can damage lipid, protein, and DNA and this will lead to oxidative stress and further tissue damage^88^. So, the ROS production in the presence of hydrogels was evaluated by NO, NBT and MDA assays. Usually, the formation of NO was measured by using Griess assay and it measures the conversion of nitrite to a purple colored azo dye^89^. Like, NBT assay is used to detect the superoxide (O_2_^−^)^90^. The literature stated that the level of MDA was higher under periodontitis conditions because of lipid peroxidation associated with oxidative stress^88^. As shown in Fig. 13, hydrogels reduced MDA, NO, and superoxide production. Local microbiota and host immune response have a crucial role in the pathogenesis of periodontitis. It has been understood that gingival fibroblasts can activate NF-κB and produce inflammatory cytokines such as IL-1β, and TNF-α^91^. IL-6 and PGE2 are known as the biomarkers for periodontitis and largely leads to bone resorption^92^. As shown in Fig. 14, the level of both of NF-κB, IL-6, and PGE2 were elivated under inflammatory condition. At the same time, the level of them were decreased after treatment with hydrogels.

For several years the hydrogels emerged as a potential drug delivery system due to its ability to mimic ECM. Pakzad and Ganji et al. developed a drug loaded thermoresponsive hydrogel composed of chitosan/gelatin/-glycerolphosphate for periodontal application. The material was biocomtible and has antimicrobial activity against *Clostridium sporogenes*^93^. An antimicrobial dental light curable bioadhesive hydrogel composed of visible-light-activated naturally derived polymer (gelatin) and an antimicrobial peptide developed by sani et al. has higher adhesion to physiological tissues and showed antimicrobial actvity against *P. gingivalis*^94^. Chen et al. developed an thermosensitive nanoparticle hydrogel for the delivery of ibuprofen and basic fibroblast growth factor. This material has significant anti-inflammatory properties and promoted the proliferation and adhesion of human gingival fibroblasts cells^95^. However, most of the studies are focused on to either material characteristics or antimicrobial/anti-inflamamtory properties. Our present study investigated the antibacterial and anti-inflammatory properties of antimicrobials loaded cellulose nanofibers and κ-carrageenan oligosaccharide composite hydrogels under periodontitis conditions. We investigated the biofilm formation, oxidative stress, and metabolic activity of bacteria along with checking the antimicrobial activity. Moreover, we investigated the anti-inflammatory properties of hydrogels in HGF cells. According to our study, the sensitivity of periodontal pathogens to the hydrogel suggesting a new alternative to conventional treatments. In addition to this, the hydrogels can reduce the inflammatory condition and possesses strong antioxidant activity. These elements are very crucial in the management of periodontitis.

## Methods

### Preparation of antimicrobials loaded hydrogels

Hydrogels were prepared according to a previously reported work with some modifications^96^. CO–CNF nanoparticles were selected for preparing drugs-loaded hydrogels. Detailed information on the preparation of CO–CNF was described in our previously published work^15^. 50 ml of CO–CNF solution was prepared by dissolving 5 g of CO–CNF in 50 ml distilled water. Each quantity of surfactin (100, 200, and 400 mg) (Sigma-Aldrich, Louis, Missouri, USA) was added to the above solution under stirring conditions (40 °C, 800 rpm) to prepare different concentrations of surfactin loaded hydrogel. The stirring was continued for 30 min. Then, 1 ml of Herbmedotcin (Giant Bio Tech, New Taipei City, Taiwan) was added to each above said system and stirred for 15 min. After, 5 ml (0.1 g/ml) of the MBAA solution was added and stirred for 2 h to obtain the complete gelation.

### Scanning electron microscopic (SEM) analysis

Morphological analysis of the hydrogels was carried out by using SEM (S-4800 Scanning Electron Microscope, HITACHI, Tokyo, Japan). Dried samples were transferred to the metal stud using double-sided tape and coated with gold using a sputter gold coater (Model-E1010 Ion sputter). Images were observed at different magnifications at an accelerating voltage of 15 kV.

### Fourier-transform infrared (FTIR) spectroscopy analysis

The FTIR spectra of the samples were studied using an FTIR spectrometer (BRUKER, TENSOR II, Massachusetts, USA) by potassium bromide (KBr) disc method. The samples were dried, ground and pelletized using KBr (1:100, w/w) to form the thin films of samples. 64 scans were performed in the range of 400–4000 cm^−1^ and the resolution was taken as 4 cm^−1^.

### Thermogravimetric analysis (TGA)

TGA instrument (NETZSCH TG 209F3, Germany) was used to evaluate the thermal degradation behavior of samples (5 mg). The temperature range was fixed at 40–600 °C with a constant heating rate of 20 °C/min under nitrogen atmosphere.

### Evaluation of swelling ratio (SR)

The swelling ratio of hydrogels was determined by immersing the dried samples in water from 0 to 12 h at room temperature. At particular points, samples were taken, remove the surface water, and subsequently transferred to measure the weight. All experiment was performed in triplicates^97^. The Swelling ratio was calculated by the following equation:

1 $$ {\text{Swelling}}\,{\text{Ratio }}\left( {{\text{SR}}} \right) \, { }\left( {\text{\%}} \right){ } = { }\frac{W\,total - W\,dry}{{W\,dry}} \times 100, $$

W dry is the weight of hydrogel in dry condition, W total is the weight of swelled hydrogel.

### DPPH radical scavenging activity

Antioxidant activities of the samples were evaluated by using DPPH radical scavenging assay according to a previously reported method^98^. 100 µl of DPPH (0.004 g in 100 ml methanol) was mixed with an equal volume of samples in a 96-well plate. The concentration of doxycycline (Swiss Pharmaceuticals Co., Ltd., Tainan, Taiwan), Herbmedotcin, and surfactin was 10 mg/ml, and the concentration of hydrogels was 40 mg/ml. Trolox (0.5 mg/ml) (Sigma-Aldrich, Louis, Missouri, USA). and methanol was selected as standard and blank, respectively. The absorbance was measured at 517 nm after 30 min of incubation period under dark.

The DPPH radical scavenging activity was calculated by the following equation:

2 $$ {\text{DPPH}}\,{\text{Radical}}\,{\text{Scavenging}}\,{\text{Activity }}\left( {\text{\%}} \right) = \frac{{{\text{A}}\,{\text{control}} - {\text{A}}\,{\text{sample}}}}{{{\text{A}}\,{\text{control}}}}{ } \times 100, $$

where, A control is the mixture of methanol and DPPH solution; A sample is the mixture of tested sample and DPPH solution.

### Entrapment efficiency (EE), loading capacity (LC), and in vitro drug release

The centrifugation method was used to evaluate the EE and LC of the hydrogel. 100 mg of surfactin was added to 50 ml of CO–CNF and cross-linked with MBAA as in the hydrogel preparation section 1 ml of the supernatant of centrifuged (3500 rpm for 10 min) hydrogel (15 mg/ml) were taken and subjected to high-pressure liquid chromatography (HPLC) (JASCO, Eaton, Maryland, USA) to evaluate EE and LC^99^. In vitro drug release was performed by enclosing the same amount of hydrogel as in EE in a dialysis bag (12 kDa) and dipped in 30 ml phosphate buffer solution (PBS) (pH 6.5) at 37 °C for 24 h under mild agitation condition (100 rpm). At each time intervals (0, 1, 2, 4, 6, 8, 12, and 24 h), 1 ml was withdrawn and replace it with fresh 1 ml PBS. Each experiment was performed in triplicates. A mixture of trifluoroacetic acid and water (0.05:99.95) (v/v) was taken as the mobile phase A and mixture of acetonitrile, water, and trifluoracetic acid (80:19.95:0.05) (v/v) was taken as mobile phase B. C18 column Phenomenex (250 mm × 4.6 mm, 5 µm) with a temperature of 30 °C was selected and the flow rate was set at 1.2 ml/min^99,100^. Samples were analyzed at 210 nm.

3 $$ EE \,  \left( \% \right) = \frac{Total\,Surfactin - Free\,Surfactin}{{Total\,Surfactin}} \times 100, $$

4 $$ LC \, \left( \% \right) = \frac{Total\,Surfactin - Free\,Surfactin}{{Total\,Weight\,of\,Hydrogel}} \times 100. $$

### Bacteria culture and ditch plate method

*S. mutans* (ATCC 25175) and *P. gingivalis* (CCUG 25211), *F. nucleatum* (ATCC 10953), *P. aeruginosa* (ATCC 9027), were purchased from Bioresource Collection and Research Center (BCRC), Hsinchu, Taiwan. The bacteria were cultured in tryptic soy broth (TSB) at 37 °C. The antimicrobial activity of samples was primarily evaluated by agar ditch plate method^101^. 100 µl of each sample was added to their respective wells made on a 10 µl bacteria (1 × 10^7^ CFU/ml) coated agar plate. The concentration of doxycycline and surfactin was 10 mg/ml and hydrogels were 40 mg/ml. The plates were incubated for 24 h at 37 °C and the zone of inhibition was measured. The experiment was performed triplicates.

### Determination of minimum inhibitory concentration (MIC) and minimum bactericidal concentration (MBC)

The MIC and MBC were determined according to a previously reported method^102^. 80 µl of each sample at different concentrations were inoculated separately with 120 μl of bacterial culture (1 × 10^5^ CFUs/ml) in a 96-well plate. The MIC was determined after overnight incubation at 37 °C. The MBC was determined by plating 100 μl of culture broth from wells onto the TSA plate and incubated at 37 °C overnight. Doxycycline was taken as the positive control. MIC and MBC were expressed in the percentage of the sample concentration.

### Inhibition of biofilm

Each sample was incubated with bacteria as mentioned in the MIC determination assay^103^. After 24 h of incubation, the wells were washed three times with 200 μl sterile distilled water and air dried for 45 min. Later, 0.1% (v/v) crystal violet solution (200 µl/ml) was added and kept it for 1 h. After washing (5 times with 300 µl sterile distilled water), solubilized the adherent dye by adding 200 μl of 95% (v/v) ethanol. The experiment was performed in triplicates. The absorbance was measured at 595 nm (ELISA Reader, Thermo Fisher 1510, Germany). The percentage inhibition of biofilm activity was calculated using the following equation:

5 $$ {\text{Inhibition}}\,{\text{of}}\,{\text{biofilm }} \, \left( {\text{\%}} \right){ } = \left[ {1 - \left( {Absorbance\,of\,cells\,treated\,with\,drugs/absorbance\,of\,non\,treated\,control\,cells} \right)} \right] \times 100. $$

### Determination of metabolic activity and oxidative stress

Incubated each sample (80 µl) sample with 120 µl of bacterial species (1 × 10^7^ CFU/ml) for 24 h at 37 °C. Metabolic activity was determined by using 3-(4,5-dimethylthiazol-2-yl)-2,5-diphenyl tetrazolium bromide (MTT). After 24 h, the medium has removed and incubated the plate with 100 µl of MTT (1 mg/ml) (Sigma-Aldrich, Louis, Missouri, USA). for 4 h. Then, 200 µl of dimethyl sulfoxide (DMSO) was added to each well (under dark condition) after the removal of MTT. The absorbance was measured at 570 nm. Oxidative stress of bacteria in the presence of hydrogels was examined by MDA assay. For MDA analysis, the treated samples were mixed with 200 µl of MDA reagent (a mixture of 47 ml water, 1 ml HCl, 7.2 g trichloroacetic acid, and 0.18 g thiobarbituric acid) and placed in a water bath (Water Bath, BUCHI 461, Zürich, Switzerland) at 100 °C for 15 min. 1,1,3,3-tetramethoxypropane was taken as the standard. After cooling, 300 µl of 1-butanol was added, shaken vigorously, and centrifuged at 1500 × *g* for 10 min. The supernatant was collected and the absorbance was measured at 532 nm^104^. The cell viability and MDA level were calculated according to the following equations:

6 $$ {\text{Bacterial}}\,{\text{viability}}\,\left( {\text{\%}} \right) = \frac{Absorbance\,of\,the\,sample - Absorbance\,of\,the\,blank}{{Absorbance\,of\,the\,control - Absorbance\,of\,the\,blank}} \times 100, $$

7 $$ {\text{MDA}}\,{\text{level }} = \frac{{Absorbance\,of\,sample\,at\,532\,{\text{nm}} - Absorbance\,of\,blank\,at\,532\,{\text{nm}}}}{{Absorbance\,of\,standard \,at \,532\,{\text{nm}} - Absorbance\,of\,blank\,at\,532\,{\text{nm}}}} \times 5. $$

### Acridine orange (AO) assay

150 µl of samples were incubated with 350 µl bacterial solution at 37 °C for 24 h. After centrifugation (3000 rpm, 3 min) and the bacteria were stained with acridine orange (1 mg/ml) for 15 min. Later, the cells were observed under a microscope^105^.

### Cell culture and determination of cell viability

Human gingival fibroblast (HGF) cells (Blossom Biotechnologies Inc., Taipei, Taiwan) were cultured in a fibroblast medium containing 10 ml fetal bovine serum (FBS), 5 ml fibroblast growth supplement, and 5 ml of penicillin/streptomycin solution (Blossom Biotechnologies Inc., Taipei, Taiwan. The cells were maintained at 37 °C in a 5% CO_2_ incubator. The toxicity of the samples was analyzed by MTT assay. The cell concentration was adjusted to 1 × 10^4^ cells/well in a 96-well plate. After 24 h of incubation, the cells were treated with 100 µl of samples (50 µg/ml) again incubated for another 24 h. Then, the cells were washed with 100 µl of PBS. Later, 100 µl of MTT reagent (1 mg/ml) was added and kept in a CO_2_ incubator for 4 h. Then, the MTT solution has removed and 200 µl of DMSO solution was added under dark condition. The absorbance was measured at 570 nm. The experiment was performed in triplicates.

### Nitroblue tetrazolium (NBT) reduction assay

The cells (2 × 10^5^ cells/well) were pre-treated with 200 µl of (50 µg/ml) samples and 100 µl of LPS (1 µg/ml) (from *Escherichia coli* O26:B6, impurities < 5% Protein) in a 12-well plate and incubated for 24 h in a CO_2_ incubator. Later, the cells were centrifuged at 800 × *g* for 15 min and the supernatant was removed. 300 µl of NBT [(1.5 mg NBT, 13.8 ml medium, 450 µl DMSO, and 750 µl Triss-buffered saline (TBS)] solution was added to the cells and incubated for 1 h in the absence of light. Centrifuged (1500 × *g*, 15 min) the obtained solution after 1 h and the supernatant was removed. 200 µl DMSO was added and the optical density was measured at 570 nm.

The percentage of inhibition of NBT reduction was calculated according to the following equation:

8 $$ {\text{Percentage}}\,{\text{of}}\,{\text{NBT}}\,{\text{reduction}}\,{\text{inhibition }}\left( {\text{\%}} \right){ } = { }\frac{A\,control - A\,sample}{{A\,control}} \times 100. $$

### Evaluation of nitric oxide and MDA production

The cell concentration was adjusted to 1 × 10^4^ cells/well in a 96-well plate. The cells were pre-treated with 20 µl of samples (50 µg/ml) and 10 µl of LPS (1 µg/ml) and incubated for 24 h in a CO_2_ incubator. After transferred 50 µl of the cultured medium into another 96-well plate and 50 µl of Griess reagent (mixture of 0.01 g sulphanilamide in 10 ml 5% phosphoric acid and 0.01 *N*-1-napthylethylenediamine dihydrochloride in 10 ml dd water) was added (kept for 10 min). Sodium nitrate (50 µl) was taken as the standard. 0.068 g sodium nitrate was dissolved in 100 ml dd water and serially diluted. The absorbance was measured at 540 nm. The MDA production was evaluated in LPS and hydrogels treated cells. Cells were treated with samples/LPS as above and 200 µl of MDA reagent (a mixture of 47 ml water, 1 ml HCl, 7.2 g trichloroacetic acid, and 0.18 g thiobarbituric acid) was added and placed in a water bath for 10 min at 100 °C. 1,1,3,3-tetramethoxypropane was taken as the standard. Later, 300 µl of 1-butanol was added and centrifuged (1500 × *g*, 10 min) after mixing. The absorbance was measured at 532 nm.

### Cytokines production

HGF cells were cultured in a fibroblast medium containing 10 ml FBS, 5 ml fibroblast growth supplement, and 5 ml of penicillin/streptomycin. Later, cells (1 × 10^4^ cells/well) were pre-treated with 20 µl of the sample (50 µg/ml) and 10 µl of LPS (1 µg/ml) and incubated for 24 h in a CO_2_ incubator. Later, samples were centrifuged (1000 × *g*, 20 min) and the supernatant was collected for evaluating transcription factor and cytokines [NF-κB (Asia Bioscience Co. Ltd., Taipei, Taiwan), IL-6, and PGE2 (Taiclone Biotech Corp., Taipei, Taiwan)] productions. All procedures were conducted according to the manufacturer’s protocol. For NF-κB determination, 100 µl standard/samples were added to respective wells and incubated at 37 °C for 1 h. After, aspirated the solution and 100 µl of detection reagent A was added, and again incubated for 1 h at 37 °C. After washing (350 µl of 1 × wash solution), incubated the plate with 100 µl detection reagent B for 30 min. Wash the plate with 5 times and incubated the plate at 37 °C with 90 µl TMB substrate solution for 15 min under dark. Later, 50 µl of stop solution was added and the liquid was turned into yellow. The absorbance was measured at 450 nm. For PGE2 determination, 50 µl of samples/standard were treated with 50 µl biotinylated detection Ab working solution and incubated for 45 min at 37 °C. After washing with 350 µl wash buffer, 100 µl of HRP conjugate working solution was added and incubated for 30 min at 37 °C. 90 µl of substrate reagent was added after washing and incubated for 15 min at 37 °C. Finally, a 50 µl stop solution was added and measured the optical density at 450 nm. For IL-6 determination, 100 of standard/samples were added to well plate and incubated for 90 min at 37 °C. 100 µl of biotinylated detection Ab reagent was added after removing the liquid and incubated at 37 °C for 1 h. After washing (3 times), 100 µl of HRP conjugate working solution was added to each well and incubated at 37 °C for 30 min. After washing (5 times), 90 µl of substrate solution was added under the dark and incubated for 15 min at 37 °C. Later, 50 µl stop solution was added measured the absorbance at 450 nm.

### Statistical analysis

The data are presented as mean ± S.D. Data were analyzed by one-way ANOVA followed by Tukey multiple comparison tests using Origin Pro 2018 SR1 b9.5.1.195 (OriginLab Corporation, Northampton, MA 01060 USA) software. p < 0.05 considered as significantly different.

## Supplementary information

Supplementary Information.

## Data Availability

The datasets generated during and/or analysed during the current study are available from the corresponding author on reasonable request.
