# Supplementary material for: Therapeutic effects of antibiotics loaded cellulose nanofiber and κ-carrageenan oligosaccharide composite hydrogels for periodontitis treatment
Source: Sci Rep. 2020 Oct 22;10:18037. doi: 10.1038/s41598-020-74845-9 (PMC7581766; doi:10.1038/s41598-020-74845-9)
Supplement: Supplementary file 1 — Supplementary Information. [file 41598_2020_74845_MOESM1_ESM.pdf]

Therapeutic Effects of Antibiotics Loaded Cellulose Nanofiber and κ-Carrageenan Oligosaccharide Composite Hydrogels for Periodontitis Treatment

Athira Johnson[1], Fanbin Kong[2], Song Miao[3], Hong-Ting Victor Lin[1], Sabu Thomas[4], Yi-Cheng Huang[1], Zwe-Ling Kong[1]*

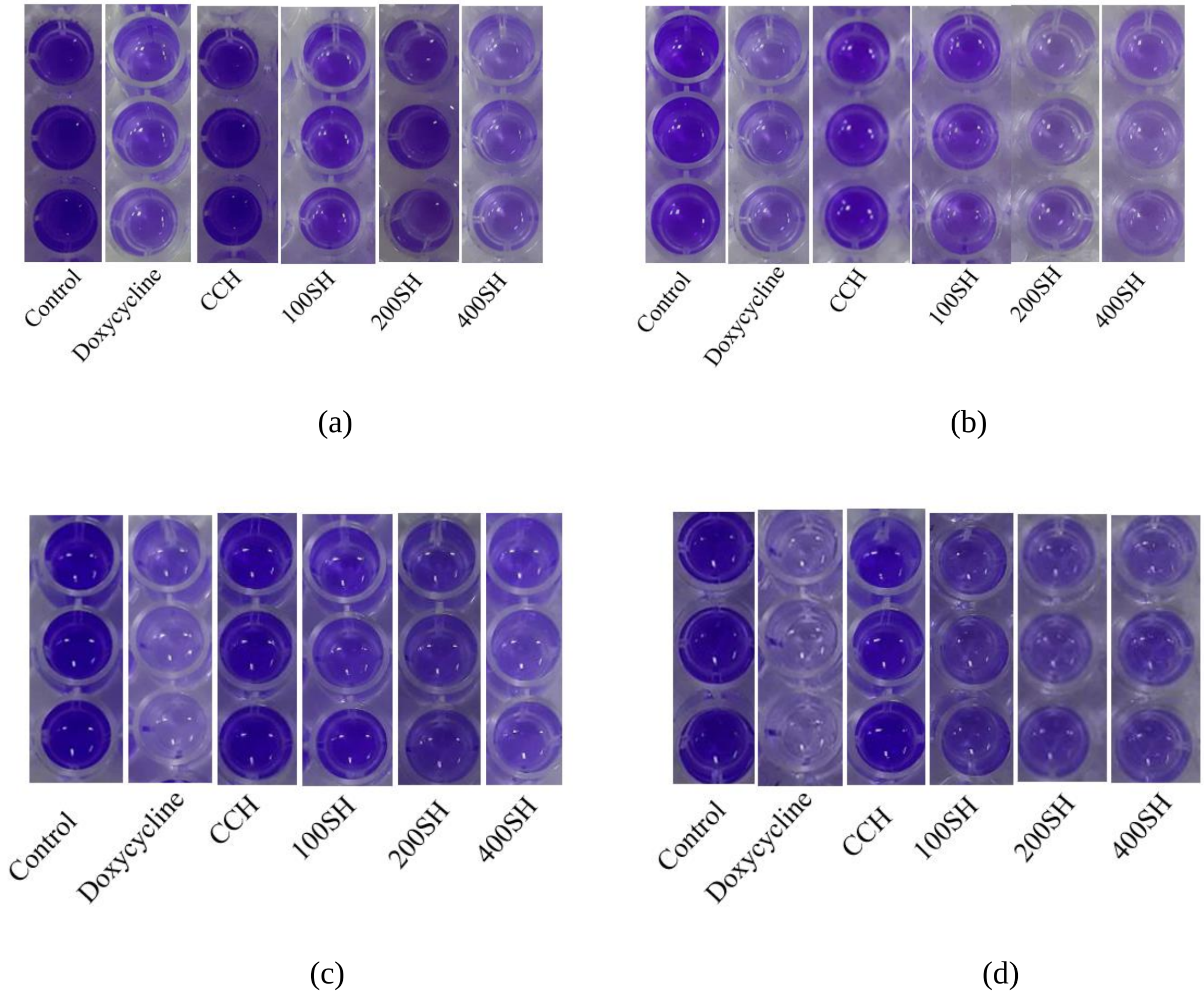

Figure S1: Visualization of the inhibition of biofilm (a) *Porphyromonas gingivalis*, (b) *Streptococcus mutans*, (c) *Fusobacterium nucleatum*, and (d) *Pseudomonas aeruginosa* by crystal violet assay. The samples were stained with 0.1% crystal violet and the absorbance was measured at 595 nm. CCH: κ- carrageenan oligosaccharides linked cellulose nanofibers

hydrogel, 100SH: 100 mg surfactin and Herbmedotcin loaded hydrogel, 200SH: 200 mg surfactin and Herbmedotcin loaded hydrogel, and 400SH: 400 mg surfactin and Herbmedotcin loaded hydrogel. Control: only bacteria.
